# Association of metformin, sulfonylurea and insulin use with brain structure and function and risk of dementia and Alzheimer’s disease: Pooled analysis from 5 cohorts

**DOI:** 10.1371/journal.pone.0212293

**Published:** 2019-02-15

**Authors:** Galit Weinstein, Kendra L. Davis-Plourde, Sarah Conner, Jayandra J. Himali, Alexa S. Beiser, Anne Lee, Andreea M. Rawlings, Sanaz Sedaghat, Jie Ding, Erin Moshier, Cornelia M. van Duijn, Michal S. Beeri, Elizabeth Selvin, M. Arfan Ikram, Lenore J. Launer, Mary N. Haan, Sudha Seshadri

**Affiliations:** 1 School of Public Health, University of Haifa, Haifa, Israel; 2 Framingham Heart Study, Framingham, MA, United States of America; 3 Department of Biostatistics, Boston University School of Public Health, Boston, MA, United States of America; 4 Department of Neurology, Boston University School of Medicine, Boston, MA, United States of America; 5 Department of Epidemiology & Biostatistics, University of California, San Francisco, California, United States of America; 6 Department of Epidemiology, Johns Hopkins Bloomberg School of Public Health, Baltimore, MD, United States of America; 7 Department of Epidemiology, Erasmus MC University Medical Center, Rotterdam, Netherlands; 8 Laboratory of Epidemiology and Population Sciences, National Institute on Aging, National Institutes of Health, Bethesda, MD, United States of America; 9 Icahn School of Medicine at Mount Sinai, New York, NY, United States of America; 10 The Joseph Sagol Neuroscience Center, Sheba Medical Center, Tel HaShomer, Israel; 11 Division of General Internal Medicine, Johns Hopkins University School of Medicine, Baltimore, MD, United States of America; 12 Department of Radiology, Erasmus University Medical Center, Rotterdam, The Netherlands; 13 Department of Neurology, Erasmus University Medical Center, Rotterdam, The Netherlands; Cardiff University, UNITED KINGDOM

## Abstract

**Objective:**

To determine whether classes of diabetes medications are associated with cognitive health and dementia risk, above and beyond their glycemic control properties.

**Research design and methods:**

Findings were pooled from 5 population-based cohorts: the Framingham Heart Study, the Rotterdam Study, the Atherosclerosis Risk in Communities (ARIC) Study, the Aging Gene-Environment Susceptibility-Reykjavik Study (AGES) and the Sacramento Area Latino Study on Aging (SALSA). Differences between users and non-users of insulin, metformin and sulfonylurea were assessed in each cohort for cognitive and brain MRI measures using linear regression models, and cognitive decline and dementia/AD risk using mixed effect models and Cox regression analyses, respectively. Findings were then pooled using meta-analytic techniques, including 3,590 individuals with diabetes for the prospective analysis.

**Results:**

After adjusting for potential confounders including indices of glycemic control, insulin use was associated with increased risk of new-onset dementia (pooled HR (95% CI) = 1.58 (1.18, 2.12);p = 0.002) and with a greater decline in global cognitive function (β = -0.014±0.007;p = 0.045). The associations with incident dementia remained similar after further adjustment for renal function and excluding persons with diabetes whose treatment was life-style change only. Insulin use was not related to cognitive function nor to brain MRI measures. No significant associations were found between metformin or sulfonylurea use and outcomes of brain function and structure. There was no evidence of significant between-study heterogeneity.

**Conclusions:**

Despite its advantages in controlling glycemic dysregulation and preventing complications, insulin treatment may be associated with increased adverse cognitive outcomes possibly due to a greater risk of hypoglycemia.

## Introduction

Dementia is a devastating clinical diagnosis that has physical, financial and social consequences for patients, their care-givers and families, including increased mortality and a greater need for medical services [1]. It is increasingly recognized that dementia is a life-course illness, preceded by years and even decades of subclinical brain changes [2], which could explain why later life disease-modifying treatments are ineffective for most people who already have dementia [3]. A major risk factor for dementia and Alzheimer’s disease (AD) is type 2 diabetes [4]. Even in persons free of clinical dementia, diabetes is associated with poor cognitive performance [5, 6] and with increased brain atrophy [5, 7].

Pharmacological treatment options for type 2 diabetes have been available for several decades, and are generally regarded as safe and well tolerated [8]. The aim of these therapies is to reduce and maintain glucose concentrations as close to normal for as long as possible after diagnosis. In turn, glycemic control is efficient in reducing micro- and macrovascular complications [9], including a modest reduction of 15% in risk of myocardial infarction and 13% reduction in all-cause mortality [10]. Yet, while type 2 diabetes may increase both AD neuropathology and cerebral infarcts in the brain [11], it is unclear whether this process can be prevented or delayed with tight glycemic control [12].

Diabetes drugs’ mechanisms of action involve multiple pathologies common to diabetes and dementia and AD, including insulin resistance and impaired glucose metabolism [13]. Thus, there is an intense interest in whether type 2 diabetes drugs can be repurposed to slow cognitive aging and reduce the risk of cognitive impairment and dementia through direct effects in the brain that are independent of their approved indications for treating high blood glucose [14]. In contrast, type 2 diabetes medications may also have detrimental effects on the brain, possibly through their tendency to cause hypoglycemic episodes [15, 16].

To date, only few clinical and observational studies have been done to assess the relationship of diabetes medications and cognitive health, and existing findings are inconsistent [17, 18]. Furthermore, it remains to be clarified whether a possible protective role is independent from the glycemic control properties of the drugs. Thus, the aim of the current study is to test whether use of insulin, sulfonylureas and metformin are associated with cognitive performance, cognitive decline, MRI measures and risk of dementia and AD, above and beyond their glycemic control properties.

## Methods

### Study population

The study is based on data from the following cohorts: The Offspring cohort of the Framingham Heart Study (FHS) [19, 20], the Rotterdam Study (RS) [21], the Atherosclerosis Risk in Communities (ARIC) Study,[22] the Aging Gene-Environment Susceptibility-Reykjavik Study (AGES) [23] and the Sacramento Area Latino Study on Aging (SALSA) [24]. The Israel Diabetes and Cognitive Decline study (IDCD) [25] contributed cross-sectional results for the cognitive function and MRI outcomes. Each of these cohorts is a large-scale, community based, longitudinal study, in which assessment of the link between impairment in glucose homeostasis and neurological outcomes is a primary goal.

The study samples included only participants with a diagnosis of diabetes. The definition of diabetes in each cohort is presented in S1 Table. In FHS, ARIC and SALSA, visits from which samples were drawn differed between the cross-sectional and prospective analyses (Table 1), because we attempted to choose the most appropriate visits for these analyses with regard to the extent of details on number of medications and duration of follow-up.

**Table 1:** Study characteristics of participants.

**Prospective analysis: incident dementia and AD (baseline characteristics)**						
	**FHS**	**AGES**	**SALSA**	**ARIC**	**RS**	**IDCD**
Year of inception	Offspring cohort exam 7 (1998–2001)	AGES II (2007–2011)	1998	Visit 4 (1996–1998)	2004–2008	N/A
N	301	623	586	1,197	608	N/A
Mean age (years)	70.1 ± 5.9	76.4±5.3	69.9±6.6	64.0±5.8	63.6±7.8	N/A
N (%) women	123 (44.4)	285 (45.8%)	313 (56.4)	646(54.0)	274 (45.1)	N/A
Duration of follow-up (years)	7.5±4.8	5.2±0.2	5.3±3.1	12.3±4.4	6.4±2.5	N/A
Incident dementia, N (%)	38 (13.7)	27 (8.1)	55 (9.4)	198 (11.24)	31 (5.1)	N/A
Incident AD, N (%) among diabetics	30 (10.8)	20 (6)	32 (5.5)	N/A	16 (2.7)	N/A
**Cross-sectional analysis: cognition**						
	**FHS**	**AGES**	**SALSA**	**ARIC**	**RS**	**IDCD**
Visit (years)	Offspring cohort exam 8 (2005–2008)	AGES I (2002–2006)	1998–1999	visit 5 (2011–2013)	2004–2008	2010–2012
N	322	694	586	1,732	451	912
Mean age (years)	70±9	77±6	70±7	76±5	63±8	73±5
N (%) women	127 (39.4)	3,166 (57)	332 (56.7)	981 (57)	199 (44)	539 (59)
**Longitudinal analysis: cognition (baseline characteristics; including prevalent dementia cases)**						
	**FHS**	**AGES**	**SALSA**	**ARIC**	**RS**	**IDCD**
Baseline visit (years)	Offspring cohort exam 7 (1998–2001)	AGES I (2002–2006)	1998–1999	Visit 4 (1996–1998)	2004–2008	N/A
N	194	287	586	1,197	250	N/A
Mean age (years)	64±9	75±4	70±7	64±6	61±7	N/A
N (%) women	80 (41)	134 (46.7)	332 (56.7)	646 (54.0)	103 (41.2)	N/A
**Cross-sectional analysis: brain MRI measures**						
	**FHS**	**AGES**	**SALSA**	**ARIC**	**RS**	**IDCD**
Visit (years)	Offspring cohort exam 8 (2005–2008)	AGES I (2002–2006)	1998–1999, 2002	visit 5 (2011–2013)	2004–2008	2010–2012
N	234	N/A	85	575	349	125
Mean age (years)	69±8	N/A	71±7	76±5	61±8	72±4
N (%) women	86 (36.8)	N/A	46 (54.1)	340 (59)	144 (41.3)	48 (38)

### Use of diabetes medications

We first assessed the distribution of medication use according to specific classes available on the market, as well as medications being used in combination (S2 Table). We focused on metformin, sulfonylurea and insulin because use of these medication classes was common at time of studies’ baseline (in contrast to other drug classes such as DPP-4 enzyme inhibitor, Meglitinide).

### Definition of dementia and AD

Information on incident dementia was available from FHS, RS, ARIC, AGES and SALSA. Incident AD was available from FHS, RS, AGES and SALSA. Dementia was defined using the Diagnostic and Statistical Manual of Mental Disorders revised third or fourth edition (DSM-IIIR or DSM-IV) criteria [f1]. AD was defined using the National Institute of Neurological and Communicative Disorders and Stroke and AD and Related Disorders Association (NINCDS-ADRDA) criteria, and included persons with definite (diagnosis of AD pathologically confirmed at autopsy), probable or possible AD [26]. Incident dementia was adjudicated in each study and was based on hospitalization, dementia diagnosed at study visits and dementia coded on the death certificate. Durations of follow-up ranged between 5.2 years (in AGES) and 12.3 years (in ARIC) (Table 1).

### MRI

Years of brain MRI examinations and number of individuals with available MRI scans in each study are presented in Table 1. MRI scans were performed and interpreted in a standardized fashion in each study, blind to subjects`clinical or demographic information. Details on MRI parameters and phenotype definition are provided elsewhere [27, 28]. Briefly, automated or semi-quantitative post-processing software was used to measure intracranial volume and total brain volume. Hippocampal volume was evaluated using operator-defined boundaries drawn on serial coronal sections or automated methods [29].

WMH burden was estimated on a quantitative scale using custom-written computer programs in AGES, FHS, and RS; in ARIC, CHS and SALSA, WMH burden was estimated on a semi-quantitative scale [30]. As well, total brain volume, hippocampal volume and white matter hyperintensity volume were expressed as percentage of intracranial volume to correct for differences in head size. White matter hyperintensity volume was log-transformed to account for skewness.

### Cognitive function

**General cognition**- Cohorts used different neuropsychological batteries. Therefore, for the current analyses, each cohort created a global cognitive score based on its available cognitive tests (S1 and S3 Tables). The global score was the first score on the unrotated principal component on a principal component analysis forcing a single score solution (PC1). Measures that had a skewed distribution were natural log-transformed, and directionality was reversed such that higher scores reflect superior performance. It has been previously shown that despite the heterogeneity in cognitive test batteries, individual differences on the general cognitive component are negligible [31]. To further validate the global cognitive components, we confirmed that their univariate associations with age, sex, education and hypertension prevalence were similar across cohorts.

**Executive function** was assessed using differences in time to complete the trails-making B and the trails-making A tests (TrB-TrA) in FHS, ARIC and IDCD. Digit span backwards was used in AGES and IDCD.

**Memory** was ascertained using word-list and paragraph recall tests. The average score on immediate and delayed recall was used as well as the delayed recall score on each test. Performance on executive function and memory were expressed as cohort specific z-scores (test scores transformed to mean zero and standard deviation one).

### Potential confounders

Educational achievement was defined as a four-class variable (no high-school degree, high-school degree only, some college and at least a college degree) in all cohorts. Physical activity was ascertained as study-specific tertiles due to heterogeneity in methodology used to assess this variable across cohorts. Smokers were those who currently smoked vs. others (former or never-smokers). Hypertension was defined as a dichotomous variable according to the JNC-8 criteria in SALSA and JNC-7 criteria in the other cohorts, with “yes” being stage 1 hypertension defined as > = 140 mmHg for systolic or > = 90 mmHg for diastolic blood pressure or on medications. Cardiovascular diseases included the following conditions: coronary heart disease, congestive heart failure, myocardial infarction, angina pectoris and coronary insufficiency. Prevalent stroke was defined as an acute onset focal neurological deficit of presumed vascular pathogenesis lasting ≥24 hours. All stroke subtypes were included except transient ischemic attacks (TIAs) (i.e. Cerebrovascular accident, atherothrombotic infarction, Cerebral Embolism, Intracerebral. Hemorrhage and Subarachnoid Hemorrhage). Body mass index (BMI) was defined by weight (in kilograms) divided by the square of height (in meters). This variable was log-transformed and used as continuous and in all but in SALSA, in which 3 categories with cutoffs at 25 and 30 were used. Depression was defined as a score of 16 or higher on the Center for Epidemiologic Studies Depression Scale (CES-D) in FHS, SALSA and RS. In ARIC, the shortened form was used hence depression was defined if score was 9 or higher. In AGES and IDCD depression was ascertained using the geriatric depression scale (GDS) with a cutoff at 10. ApoE4 carriership was defined as having at least one ε4 allele. Glycemic control indices were chosen as follows: Hemoglobin A1C (HbA1C) was used in FHS, AGES and ARIC studies, but was not available in SALSA (at baseline) or in RS. Thus, blood glucose was used as a measure of glycemic control. Tests for blood glucose were done in fasting and random states in SALSA and RS, respectively.

### Statistical analysis

Users of each of metformin, sulfonylurea and insulin drug classes, as a single therapy or in combination with other treatments, were compared to non-users of the specific class. All analyses were performed separately in each cohort and then pooled using meta-analytical techniques.

In the cross-sectional analyses, we assessed the relationships of each of metformin, sulfonylurea and insulin use with global (PC1), domain-specific cognitive scores and brain MRI measures using linear regression models. Any measure with skewed distribution was log-transformed and directionality was reversed such that higher scores reflect better performance.

The associations between medication use and change in global cognitive function were assessed using linear mixed models, with random slope and intercept, and including an interaction term between the treatment group and time between cognitive evaluations. In each cohort, participants were included if they had two cognitive assessments or more. Cognitive change was assessed using the difference between two PC1 measurements: the first was evaluated from a baseline visit and the second was the last PC1 available from follow-up examinations. Although information on more than two cognitive evaluations was available for most studies, we chose to use only the first and last ones to avoid bias due to multiple cognitive testing among individuals who are suspected for cognitive impairment. Follow-up PC1 was standardized using the same mean and standard deviation as the baseline PC1 to ensure that changes in standardized PC1 were due to changes in cognition and not due to differences in the mean and standard deviation between baseline and follow-up. All analyses were first conducted including individuals with prevalent dementia, and then excluding them in secondary analyses.

The relationships between diabetes treatment and incident dementia and AD were assessed using multivariable Cox proportional hazard models using time on study as the time scale. For these analyses, each study excluded prevalent dementia/AD at baseline.

Models were adjusted first for age, sex, education (except for MRI outcomes), interval between exam cycles and cognitive/MRI examination (except for cognitive change outcomes), then additionally for physical activity, hypertension, cardiovascular disease, stroke, total cholesterol, smoking, depression, and BMI. In a subsequent model we also controlled for HbA1C or fasting or random blood glucose (depending on cohort-specific data availability) and ApoE4. To reduce risk for indication bias we conducted several secondary analyses as follows: first, we excluded subjects with DM who do not take DM medications. Second, post-hoc analyses were done to assess the relationship of further potential confounders with DM medication use. eGFR was found to be strongly associated with indication in most studies. Therefore, the models relating DM drug class to incident dementia and AD have also been adjusted for eGFR. Lastly, we added diabetes duration as another potential confounder in our models, but this analysis was restricted to participants from the ARIC study where this variable was available.

### Meta-analysis

Study-specific beta-estimates and log hazard ratios (later exponentiated) were combined into pooled values with 95% confidence intervals. The I^2^ statistic, representing the percentage of the variability in risk estimates that is caused by heterogeneity rather than chance was employed to quantify heterogeneity [32]. Summary results were thought to be substantial heterogeneity if I^2^>0.75. In the presence of low heterogeneity, we used fixed-effect models; however, random-effect models, which consider heterogeneity across cohorts and consequently yield more conservative pooled results were additionally performed as a secondary analysis.

## Results

The total number of participants was 3,590 in the prospective dementia/AD analysis, 4,697 and 2,514 were available for the cross-sectional and longitudinal cognitive performance analyses, respectively, and 1,243 were available for the brain MRI outcomes. Participant characteristics are presented for each cohort and separately for the prospective and cross-sectional analyses (Table 1 and S4 Table). Mean ages ranged between 64±8 years (in RS) and 76±5 years (AGES) for incident dementia outcomes, between 63±8 years (in RS) and 77±6 years (AGES) for the cognitive outcomes and between 61±8 years (in RS) and 76±5 years (in ARIC) for the MRI outcomes (Table 1).

### Incident dementia and AD

Overall, formal tests for heterogeneity showed no statistically significant heterogeneity across cohorts (S5 Table). Therefore, fixed-effect models were primarily used to pool Hazard Ratios (HRs).

**Fig 1:**
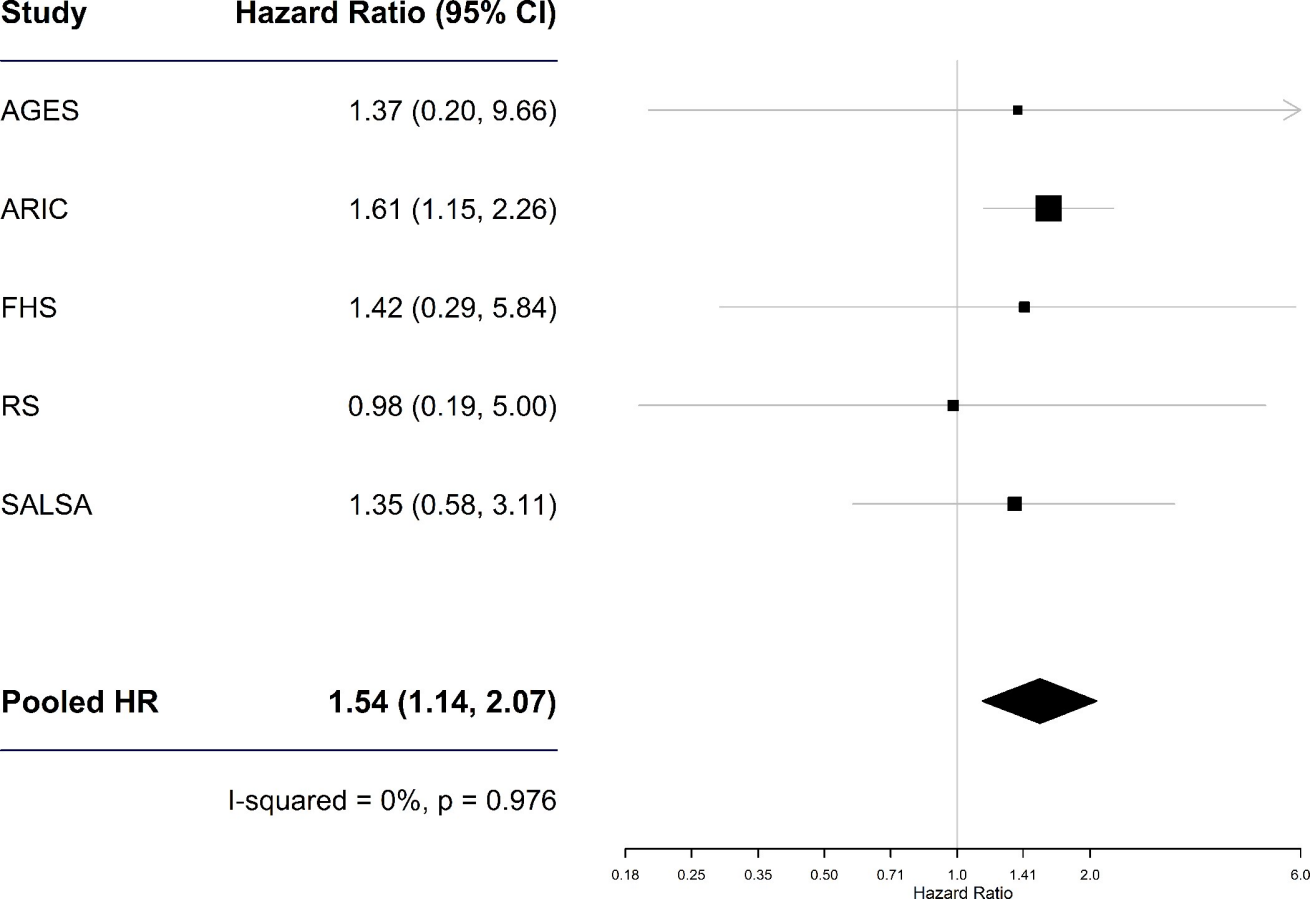
Association between insulin use and dementia risk. Comparison was made between users of insulin as a single drug or in combination with other diabetic drugs and non-users of insulin.

Compared to individuals with diabetes who did not use insulin, those who did had an increased risk for dementia independently of multiple potential confounders, including depression and HbA1C or Glucose levels (HR(95% CI) = 1.58 (1.18, 2.12); p = 0.002, Table 2, model 3 and Fig 1). No significant associations between insulin use and AD risk were identified. The associations between insulin use and incident dementia remained significant after additional adjustment for eGFR (HR (95% CI) = 1.54 (1.14, 2.07) (Table 2), after excluding from the comparison group individuals with diabetes who were not on any diabetes medication (HR (95% CI) = 1.49 (1.07, 2.07) (S6A Table) and when random rather than fixed-effects meta-analysis was used (HR (95% CI) = 1.54 (1.14, 2.07) and HR (95% CI) = 1.55 (1.12, 2.15) among diabetes patients and users of diabetes medications, respectively (S7A and S7B Table). In analyses restricted to participants from the ARIC study, additional adjustment for diabetes duration attenuated the associations: HRs (95% CI) went down from 1.61 (1.15,2.26) to 1.31 (0.90,1.92) for all persons with diabetes, and from 1.42 (0.98,2.07) to 1.34 (0.89,2.01) for those who received diabetes treatment.

**Table 2:** Associations of diabetes drug classes (single or in combination) with risk of dementia/AD among individuals with diabetes- fixed effects meta-analysis.

		Metformin		Sulfonylurea		Insulin	
Outcome	# cohorts	HR (95% CI)	p-value	HR (95% CI)	p-value	HR (95% CI)	p-value
**Model 1**							
Incident AD	4	1.37 (0.83, 2.27)	0.222	0.91 (0.59, 1.41)	0.677	1.61 (0.90, 2.89)	0.112
Incident Dementia	5	1.26 (0.94, 1.7)	0.125	0.97 (0.78, 1.22)	0.800	1.61 (1.23, 2.11)	**<0.001**
**Model 2**							
Incident AD	4	1.62 (0.92, 2.85)	0.097	0.98 (0.6, 1.6)	0.927	1.42 (0.67, 3)	0.358
Incident Dementia	5	1.35 (0.98, 1.85)	0.065	0.97 (0.77, 1.23)	0.828	1.56 (1.17, 2.08)	**0.002**
**Model 3**							
Incident AD	4	1.61 (0.89, 2.9)	0.116	1.04 (0.62, 1.74)	0.871	1.28 (0.56, 2.93)	0.556
Incident Dementia	5	1.36 (0.98, 1.89)	0.063	0.98 (0.77, 1.24)	0.853	1.58 (1.18, 2.12)	**0.002**
**Model 4**							
Incident AD	4	1.60 (0.87, 2.93)	0.131	0.9 (0.52, 1.57)	0.712	1.24 (0.53, 2.88)	0.616
Incident Dementia	5	1.42 (1.02, 1.98)	**0.038**	0.98 (0.77, 1.26)	0.894	1.54 (1.14, 2.07)	**0.005**

AD = Alzheimer’s Disease. Model 1 is adjusted for age, sex and education. Model 2 is additionally adjusted for Physical activity, hypertension, CVD, stroke, total cholesterol, smoking, depression and BMI. Model 3 is additionally adjusted for HbA1C, ApoE4. Model 4 is additionally adjusted for eGFR.

Overall, sulfonylurea use vs. non-use was not significantly associated with risk for dementia or AD (Table 2 and S6A, S7A and S7B Tables). An exception was a decreased dementia risk associated with sulfonylurea use but only when the sample was restricted to those who take diabetes medications (HR (95% CI) = 0.64 (0.46, 0.88); p = 0.007) (S6A Table; model 4), and in the fixed but not the random effect models (S7A and S7B Table). Risk of dementia among Metformin users compared to non-users was increased, however statistically significance was apparent only after adjustment for the study’s covariates including kidney function (HR (95% CI) = 1.42 (1.02, 1.98); p = 0.038) (Table 2; model 4).

### Cognitive performance, cognitive change and brain MRI measures

After adjustment for the study’s covariates, no significant association was observed between metformin, sulfonylurea or insulin use and global or test-specific cognitive function (Table 3 and S6B, S7C and S7D Tables).

**Table 3:** Associations of diabetes drug classes (single or in combination) with cognitive performance among individuals with diabetes- fixed effects meta-analysis (dementia cases are included).

		Metformin			Sulfonylurea			Insulin		
Outcome	# cohorts	Estimate	SE	p-value	Estimate	SE	p-value	Estimate	SE	p-value
**Model 1**										
Global cognition	6	-0.030	0.027	0.256	-0.075	0.029	**0.010**	-0.190	0.038	**<0.001**
Executive function (trails B-A)	3	-0.043	0.036	0.238	-0.079	0.044	0.070	-0.110	0.055	**0.047**
Executive function (digit span backwards)	2	-0.077	0.053	0.148	-0.078	0.061	0.206	-0.036	0.102	0.721
Word list - delayed	5	-0.013	0.029	0.664	-0.027	0.032	0.393	-0.084	0.041	**0.043**
Word list - combined	4	-0.046	0.039	0.242	-0.019	0.041	0.644	-0.003	0.059	0.956
Paragraph recall - delayed	3	0.042	0.034	0.226	-0.012	0.040	0.760	-0.076	0.049	0.117
Paragraph recall - combined	3	0.057	0.034	0.097	0.003	0.040	0.941	-0.075	0.049	0.126
**Model 2**										
Global cognition	6	-0.049	0.027	0.068	-0.073	0.029	**0.014**	-0.110	0.038	**0.004**
Executive function (trails B-A)	3	-0.044	0.037	0.237	-0.072	0.045	0.113	-0.085	0.058	0.144
Executive function (digit span backwards)	2	-0.077	0.054	0.154	-0.047	0.066	0.476	0.012	0.103	0.907
Word list - delayed	5	-0.030	0.030	0.317	-0.019	0.033	0.555	-0.054	0.043	0.216
Word list - combined	4	-0.037	0.039	0.348	0.002	0.041	0.965	0.069	0.059	0.245
Paragraph recall - delayed	3	0.019	0.036	0.592	-0.042	0.043	0.325	-0.036	0.053	0.499
Paragraph recall - combined	3	0.034	0.036	0.339	-0.025	0.042	0.548	-0.029	0.053	0.579
**Model 3**										
Global cognition	6	-0.030	0.027	0.274	-0.045	0.030	0.137	-0.034	0.041	0.400
Executive function (trails B-A)	3	-0.031	0.040	0.445	-0.050	0.049	0.304	-0.005	0.063	0.933
Executive function (digit span backwards)	2	-0.087	0.054	0.106	-0.028	0.066	0.671	0.051	0.108	0.637
Word list - delayed	5	-0.027	0.031	0.379	-0.011	0.034	0.739	-0.019	0.046	0.676
Word list - combined	4	-0.046	0.040	0.247	-0.005	0.042	0.908	0.075	0.060	0.215
Paragraph recall - delayed	3	0.024	0.037	0.507	-0.036	0.045	0.418	0.002	0.058	0.974
Paragraph recall - combined	3	0.036	0.036	0.328	-0.019	0.044	0.659	0.007	0.058	0.902

Model 1: Age, sex, education and interval between exam cycle and the NP assessment. Model 2: Model 1 + Physical activity, hypertension, CVD, stroke, total cholesterol, smoking, depression, BMI. Model 3: Model 2+HbA1C/ fasting blood glucose /random state blood glucose and ApoE4.

Some evidence of a greater decline in global cognitive performance was observed in those who use sulfonylurea compared to those who use other medications or life-style change (Table 4). However, these associations were no longer significant after excluding individuals with prevalent dementia at baseline (Table 4), after excluding those who are on life-style change only (S6C Table) or when random effect models were used (S7E and S7F Table).

**Table 4:** Associations of diabetes drug classes (single or in combination) with change in global cognition among individuals with diabetes- Fixed effects meta-analysis.

		Metformin*			Sulfonylurea			Insulin		
Model	# cohorts	Estimate	SE	p-value	Estimate	SE	p-value	Estimate	SE	p-value
Including prevalent dementia										
1	5	0.005	0.007	0.526	-0.02	0.005	**0.047**	-0.007	0.007	0.269
2	5	0.004	0.008	0.634	-0.011	0.005	**0.043**	-0.009	0.007	0.197
3	5	0.004	0.008	0.650	-0.012	0.005	**0.034**	-0.011	0.007	0.132
Excluding prevalent dementia										
1	5	0.003	0.007	0.728	-0.007	0.005	0.200	-0.012	0.007	0.068
2	5	0.002	0.008	0.771	-0.008	0.005	0.160	-0.013	0.007	0.062
3	5	0.002	0.008	0.827	-0.009	0.006	0.109	-0.014	0.007	**0.045**

Model 1: age, sex and education. Model 2: further adjustment for physical activity, hypertension, CVD, stroke, total cholesterol, smoking, depression, and BMI. Model 3: Further adjustment for HbA1C/ fasting blood glucose /random state blood glucose and ApoE4.

Lastly, a significant association was identified between sulfonylurea use and smaller total brain volume after adjusting for potential confounders (β = -0.007±0.003; p = 0.037) (Table 5). Nevertheless, these associations were no longer significant after excluding individuals with prevalent dementia (Table 5), after excluding participants with diabetes who do not take diabetes medications (S6D Table), and when random effect meta-analyses were used (S7G and S7H Table).

**Table 5:** Associations of diabetes drug classes (single or in combination) with brain MRI measures among individuals with diabetes- Fixed effects meta-analysis.

			Metformin			Sulfonylurea			Insulin		
Model	Outcome	# cohorts	Estimate	SE	p-value	Estimate	SE	p-value	Estimate	SE	p-value
Including prevalent dementia											
1	TCBV	5	-0.003	0.002	0.189	-0.010	0.003	**<0.001**	-0.012	0.004	**0.005**
	HPV	5	0.00001	0.00001	0.318	0.000003	0.00001	0.766	-0.00002	0.00002	0.315
	WMHV	6	0.062	0.038	0.105	0.077	0.044	0.083	0.144	0.060	**0.016**
2	TCBV	5	-0.002	0.002	0.389	-0.008	0.003	**0.014**	-0.011	0.004	**0.011**
	HPV	5	0.00001	0.00001	0.318	0.000002	0.00001	0.843	-0.00002	0.00002	0.315
	WMHV	6	0.037	0.0382	0.339	0.046	0.045	0.301	0.062	0.058	0.280
3	TCBV	5	-0.001	0.002	0.632	-0.007	0.003	**0.037**	-0.008	0.004	0.054
	HPV	5	0.00001	0.00001	0.318	0.00001	0.00001	0.318	-0.00001	0.00002	0.615
	WMHV	6	0.030	0.039	0.444	0.034	0.045	0.447	0.040	0.060	0.509
Excluding prevalent dementia											
1	TCBV	4	-0.005	0.003	0.0915	-0.011	0.003	**<0.001**	-0.009	0.006	0.154
	HPV	4	-0.002	0.005	0.650	-0.006	0.005	0.255	-0.011	0.006	0.090
	WMHV	5	0.0467	0.040	0.241	0.078	0.046	0.089	0.151	0.062	**0.014**
2	TCBV	4	0.004	0.003	0.188	-0.007	0.004	0.075	-0.006	0.006	0.357
	HPV	4	-0.004	0.005	0.404	-0.005	0.006	0.396	-0.013	0.007	0.080
	WMHV	5	0.0186	0.040	0.640	0.049	0.046	0.286	0.074	0.059	0.210
3	TCBV	4	-0.002	0.003	0.500	-0.008	0.006	0.170	-0.003	0.006	0.578
	HPV	4	-0.002	0.005	0.6712	-0.003	0.006	0.636	-0.011	0.008	0.168
	WMHV	5	0.012	0.041	0.775	0.041	0.046	0.374	0.0617	0.061	0.316

TCBV = Total cerebral brain volume; HPV = Hippocampal volume; WMHV = White matter hyperintensity volume. Model 1: age, sex and education. Model 2: further adjustment for physical activity, hypertension, CVD, stroke, total cholesterol, smoking, depression, and BMI. Model 3: Further adjustment for HbA1C/ fasting blood glucose /random state blood glucose and ApoE4.

## Discussion

The main findings from this 5-cohorts pooled analysis of 3,590 individuals with diabetes, is that using insulin was associated with 50% increased dementia risk compared to using other treatments for diabetes. In addition, metformin and sulfonylurea use was not associated with dementia risk nor with other measures of cognitive aging.

Administration of exogenous insulin (through controlled infusion while maintaining constant glucose levels [33] or through intranasal administration) has been suggested as a promising therapeutic approach against dementia and AD. Particularly, intranasal administration of insulin has shown promising results in slowing brain aging and improving cognitive function among demented individuals [34, 35], although various modifying effects such as by ApoE genotype and dosing need to be elucidated [36, 37]. These findings are supported by basic research, showing that insulin exerts various neuromodulatory actions in the brain with implications on cognitive function and neurodegeneration, including synaptic formation and remodeling, regulation of neurotransmitters, amyloid clearance, and tau phosphorylation [38]. In contrast to these neuroprotective effects, peripheral insulin administration to achieve glycemic control in diabetic patients may have distinct consequences. In line with our findings, a recent case-control study demonstrated a positive association between insulin use and dementia risk [18]. Peripheral insulin use may result in deleterious effects to the brain, due to its tendency to induce hypoglycemia. Indeed, episodes of hypoglycemia has been long associated with increased risk of dementia in many [16, 39], although not all [40, 41] studies. In the prospective population-based Health, Aging, and Body Composition study, a bidirectional association has been demonstrated between hypoglycemia and dementia risk among 783 older adults, with an estimated 2-fold increase in dementia risk among individuals who experienced hypoglycemic episodes compared to those who did not [42]. Postulated underlying mechanisms include metabolic insult as a consequence of brain mitochondrial dysfunction and increased oxidative stress in the brain [43–45]. Although information on hypoglycemic episode was not available in our samples, others have shown that the overall incidence of hypoglycemia requiring medical intervention among adults with type 2 diabetes is considerable, and is strongly linked with insulin use [46]. Numbers of hypoglycemic episodes is much larger if mild-to-moderate episodes are considered [47], however the extent of their association with dementia risk is unclear.

Insulin use was associated with risk of dementia but not AD. In addition, the attenuation in effect sizes after controlling for potential covariates was greater when the outcome was incident AD rather than incident dementia. This may indicate that vascular mechanisms underlie these findings, as vascular dementia is the second most frequent dementia subtype after AD [48]. Yet, it is important to note that results from the ARIC study were not included in the pooled AD risk estimate, which may decrease statistical power to detect such an association.

In our meta-analysis results, metformin and sulfonylurea were not associated with measures of brain function and structure. Metformin, a Biguanide, reduces insulin-mediated hepatic glucose production and increases peripheral glucose disposal [49]. In the context of AD, metformin has been suggested as a potentially anti-AD treatment, partly due to its roles in neuroprotection, in decreasing insulin resistance and prevention of AD-like pathological characteristics [50, 51]. However, determinantal effects in terms of AD risk have also been demonstrated in pre-clinical studies, where exacerbation of AD pathology has been shown [52] together with possible mechanisms affecting brain damage [53]. Similarly, findings from epidemiologic research are conflicting, with some showing decreased risk of cognitive decline [54] and dementia [55, 56] as well as improved cognitive performance [57, 58], while others demonstrating no association of metformin use with cognitive outcomes [59] as in our study, or even slightly increased AD risk [60].

Compared to metformin, mechanisms of sulfonylureas are less clear in general and particularly in the context of brain health [61]. In addition, the associations of sulfonylurea with cognitive outcomes have been rarely studied. Overall, we did not find associations with brain health, which is consistent with most existing studies showing no associations with cognitive function [57] and dementia risk [60, 62]. In contrast, we found some evidence of protective effect when comparing sulfonylurea users to others who receive diabetic medications (excluding those on life-style change), in line with several other studies that suggest a neuroprotective effect for sulfonylurea [55, 56].

The inconsistency between our findings and previous literature may stem from heterogeneity in study population, design and methodologies. One methodological difference worth noting is the lack of some studies to adjust for measures of glycemic control [56, 57, 60], which impair their ability to infer on the role of diabetes medication use *per se* (i.e. above and beyond their role in controlling blood glucose levels). A recent study among elderly US veterans compared dementia risk in 17,200 new users of metformin to 11,440 new users of sulfonylurea, and found lower risk among metformin users in a subsample of veterans aged <75 years [62]. This study was retrospective, utilizing data from national Veterans Administration clinical and administrative databases and Medicare, and therefore lacked information on education and was prone to misclassification of key measures including dementia incidence and to ascertainment bias. In contrast, our study combined data from prospective, population-based cohorts, each of which carefully ascertained dementia cases and other important clinical and demographic information and may have better representation of the general population.

Observational studies are essential in assessing the link between medical treatments and long-term cognitive health [14, 63]. While randomized controlled trials (RCTs) are considered the best levels of evidence and are the only study design which can establish causality, their role in understanding the relationships between diabetes medications and cognitive outcomes is limited. Among other weaknesses, RCTs are often restricted by head-to-head comparisons and short follow-up duration resulting in insufficient power to detect changes in cognitive function or assessment of incipient dementia cases. Observational study designs can overcome some of these problems and are “closer” to real world in terms of the heterogeneity of the study sample. A major threat to observational studies assessing the comparative role of various treatments on disease prevention is confounding by indication. In our study, the possibility that our finding of increased dementia risk among insulin users compared to non-users is a consequence of such bias cannot be excluded, as insulin treatment is usually given in advanced phases of the disease, after life-style change and oral medications are no longer effective in controlling of blood glucose [64]. Indeed, among the participants from the ARIC study, the associations between insulin use and dementia risk attenuated after additional adjustment for diabetes duration. Nevertheless, the association of insulin use with dementia risk in the total sample remained robust even after excluding individuals who are in their early phases of the disease (not treated with medications), and after adjusting for measures of glycemic control and eGFR. Of note, the latter is an important covariate as renal function correlates with duration of diabetes [65] and affects diabetes drug choice [66]. These results, together with the biological rationale of hypoglycemic episodes influences, imply that the increased dementia risk among insulin users cannot be fully explained by indication bias.

Other limitations of the study are as follows: First, most participating cohorts did not have data on diabetes duration, and therefore this variable was not included as a covariate. However, diabetes duration is strongly correlated with eGFR [65] which was adjusted for in our models. In addition, we were not able to assess the relationship of newer diabetes medication classes with the study’s outcomes, as calendar times of assessments go back to times when these treatments were not available. Lastly, individuals from the participating cohorts are predominantly of European ancestry, yet it should be noted that ARIC study, which includes ~25% African-Americans, drives much of the association between insulin use and incident dementia.

The study has several strengths worth mentioning: first, by pooling data from five large cohorts we created a large group of individuals with prospectively ascertained diabetes, thus optimized our power to detect associations which may otherwise could not be identified. In addition, careful harmonization of variables between cohorts was conducted, and data was analyzed according to pre-specified statistical analysis plans, which helped reduce heterogeneity across cohort-specific results. In addition, in contrast to data-pooling from published works, our findings are not subjected to publication bias. Lastly, we adjusted for potential confounders including markers of disease severity and glycemic control, therefore we could assess the possible roles of treatments in cognitive health beyond their glycemic control effects and reduced the possibility of confounding by indication.

Our findings raise concern regarding increased dementia risk among middle-aged and old-adults who use insulin. Future research is encouraged to investigate the possible mediation role of hypoglycemic episodes in this association, and to identify modifiers which will enable more personalized diabetes treatment to reduce dementia risk.

## Supporting information

S1 Table Diabetes definition by cohort. (PDF)

S2 Table Distribution of Medications. S2a Table: Cohort-specific sample distribution by diabetes treatment
S2b Table: Cohort-specific sample distribution by number of medications.
(PDF)

S3 Table Cognitive tests used to create PC1 for global cognition in each cohort. (PDF)

S4 Table Descriptives. S4a Table: Baseline characteristics of FHS study participants by history of diabetes: prospective analyses of incident dementia/AD
S4b Table: Baseline characteristics of FHS study participants: longitudinal analyses of change in cognition (including prevalent dementia)
S4c Table: Baseline characteristics of FHS study participants by history of diabetes: cross-sectional analyses
S4d Table: Baseline characteristics of AGES study participants by diabetes status: prospective analyses
S4e Table: Baseline characteristics of AGES study participants: longitudinal analyses of change in cognition
S4f Table: Baseline characteristics of AGES study participants by diabetes status: cross-sectional analyses
S4g Table: Baseline characteristics of SALSA study participants by diabetes status: prospective analyses
S4h Table: Baseline characteristics of SALSA study participants: longitudinal analyses of change in cognition
S4i Table: Baseline characteristics of SALSA study participants by diabetes status: cross-sectional analyses
S4j Table: Baseline characteristics of ARIC study participants by diabetes status: prospective analyses
S4k Table: Baseline characteristics of ARIC study participants: longitudinal analyses of change in cognition
S4l Table: Baseline characteristics of ARIC study participants by diabetes status: cross-sectional analyses
S4m Table: Baseline characteristics of RS study participants by diabetes status: prospective analyses
S4n Table: Baseline characteristics of RS study participants: longitudinal analyses of change in cognition
S4o Table: Baseline characteristics of RS study participants by diabetes status: cross-sectional analyses
S4p Table: Baseline characteristics of IDCD study participants by diabetes status: cross-sectional analyses.
(PDF)

S5 Table Assessment of Heterogeneity. S5a Table: Heterogeneity statistics for the associations of diabetes drug classes with incident dementia and AD among individuals with diabetes
S5b Table: Heterogeneity statistics for the associations of diabetes drug classes with cognitive performance among individuals with diabetes
S5c Table: Heterogeneity statistics for the associations of diabetes drug classes with cognitive change among individuals with diabetes
S5d Table: Heterogeneity statistics for the associations of diabetes drug classes with brain MRI measures among individuals with diabetes.
(PDF)

S6 Table Analysis among a subsample of participants with diabetes who take diabetes medications (excluding those who are on life-style change only). S6a Table: Associations of diabetes drug classes with risk of dementia/AD among individuals with diabetes who receive diabetes medications
S6b Table: Associations of diabetes drug classes with cognitive performance among individuals with diabetes who receive diabetes medications
S6c Table: Associations of diabetes drug classes with change in global cognition among individuals with diabetes who receive diabetes medications
S6d Table: Associations of diabetes drug classes with brain MRI measures among individuals with diabetes who receive diabetes medications.
(PDF)

S7 Table Random effect meta-analyses. S7a Table: Associations of diabetes drug classes with incident dementia/AD among individuals with diabetes
S7b Table: Associations of diabetes drug classes with incident dementia/AD among diabetic participants who are on medications (excluding those who are only on life-style change)
S7c Table: Associations of diabetes drug classes with cognitive performance among individuals with diabetes
S7d Table: Associations of diabetes drug classes with cognitive performance among individuals with diabetes who are on medications (excluding those who are only on life-style change)
S7e Table: Associations of diabetes drug classes with change in cognitive performance among individuals with diabetes
S7f Table: Associations of diabetes drug classes with change in cognitive performance among individuals with diabetes who are on medications (excluding those who are only on life-style change)
S7g Table: Associations of diabetes drug classes (single or in combination) with MRI measures among individuals with diabetes
S7h Table: Associations of diabetes drug classes (single or in combination) with MRI measures among individuals on diabetes medications.
(PDF)
