# Supplementary material for: Association of metformin, sulfonylurea and insulin use with brain structure and function and risk of dementia and Alzheimer’s disease: Pooled analysis from 5 cohorts
Source: PLoS One. 2019 Feb 15;14(2):e0212293. doi: 10.1371/journal.pone.0212293 (PMC6377188; doi:10.1371/journal.pone.0212293)
Supplement: S1 Table — (PDF) [file pone.0212293.s001.pdf]

S1 Table. Diabetes definition by cohort

Cohort	definition
**FHS**	Random blood glucose ≥ 200 mg/dL or fasting blood glucose ≥ 126 or on diabetes medication
**SALSA**	self-report of a physician's diagnosis, fasting blood glucose ≥ 126, or on diabetes medication
**ARIC**	self-report of a physician diagnosis or glucose lowering medication use
**RS**	General practitioners' records (including laboratory glucose measurements), hospital discharge letters, and serum glucose measurements from the Rotterdam Study visits
**AGES**	Self-report of a physician's diagnosis, use of diabetes medication, or fasting glucose≥ 7mmol/l
**IDCD**	Self-report of a physician's diagnosis, use of diabetes medication, fasting glucose>125 or HbA1c>=6.5
