# Supplementary material for: Association of metformin, sulfonylurea and insulin use with brain structure and function and risk of dementia and Alzheimer’s disease: Pooled analysis from 5 cohorts
Source: PLoS One. 2019 Feb 15;14(2):e0212293. doi: 10.1371/journal.pone.0212293 (PMC6377188; doi:10.1371/journal.pone.0212293)
Supplement: S2 Table — S2a Table: Cohort-specific sample distribution by diabetes treatment S2b Table: Cohort-specific sample distribution by number of medications. (PDF) [file pone.0212293.s002.pdf]

S2. Distribution of medications

S2a Table: Cohort-specific sample distribution by diabetes treatment

	**FHS**	**AGES**	**SALSA**	**ARIC**		**RS**	**ICDC**
**Treatment**	N=322	N=694	N=586	N=1,197 (Prospective analysis)	N=1732 (cross-sectional analysis)	N=451	N=912
Life-style change *, n (%)	130 (40.4)	346 (49.9)	212 (36.2)	141 (11.2)	670 (38.3)	312 (50.8)	149 (17)
Insulin (Ins)	33 (10.3)	37 (5.3)	95 (16.2)	387 (32.3)	276 (15.7)	78 (12.7)	79 (9)
Sulfonylurea (SU)	104 (32.3)	204 (29.4)	271 (46.3)	580 (48.5)	418 (23.8)	126 (20.5)	289 (33)
Metformin (Met)	123 (38.2)	226 (32.6)	135 (23.0)	183 (15.3)	691 (39.3)	160 (26.1)	702 (80)
Thiazolidinedione (TZD)	48 (14.9)	38 (5.5)	16 (2.7)	51 (4.3)	76 (4.3)	13 (2.1)	83 (9)
d-Phenylalanine derivative	1 (0.3)	0 (0)	0 (0)	0 (0)	0 (0)	NA	0 (0)
Meglitinide	0 (0)	8 (1.2)	0 (0)	0 (0)	3 (0.2)	NA	158 (18)
Alpha-glucosidase inhibitor	1 (0.3)	1 (0.1)	3 (0.5)	17 (1.4)	2 (0.1)	NA	103 (12)
DPP-4 enzyme inhibitor	0 (0)	0 (0)	0 (0)	0 (0)	109 (6.2)	NA	192 (22)
Other medications	5 (1.6)	0 (0)	7 (1.2)	0 (0)	4 (0.2)	50 (8.1)	178 (20)

Numbers are N (%)

*Life-style change is percentage of subjects with diabetes but not on diabetic medication

Number of total sample is based on samples assessed for cognitive function; Each medication could be used solely or in combination with other drugs

S2b Table: Cohort-specific sample distribution by number of medications

	Combinations	FHS	AGES	SALSA	ARIC	RS	ICDC
Single drug	Total	95 (29.5)	203	236 (40.3)	842	312	
	Ins	17 (17.9)	12 (1.7)	42 (7.2)	188 (22.2)	44 (22.7)	8 (0.9)
	SU	32 (33.7)	84 (12.1)	150 (25.6)	179 (21.2)	52 (26.8)	6 (0.7)
	Met	37 (39.0)	107 (15.4)	38 (6.5)	427 (50.7)	61 (31.4)	276 (31)
	TZD	9 (9.5)	N/A	2 (0.3)	15 (1.8)	0 (0)	0 (0)
	Other	0 (0)	6 (0.9)	4 (0.7)	33 (3.9)	37 (19.1)	4
2 drugs combinations	Total	71 (22.0)	116 (16.7)	124 (21.2)	381	92	229 (26)
	Ins/Met	6 (8.5)	5 (0.7)	10 (1.7)	77 (20.2)	23 (25.0)	20 (9)
	Ins/SU	2 (2.8)	7 (1.0)	27 (4.6)	34 (8.9)	7 (7.6)	2 (1)
	Ins/TZD	1 (1.4)	N/A	4 (0.7)	2 (0.5)	0 (0)	0 (0)
	Ins/other	0 (0)	1 (0.1)	1 (0.2)	0 (0)	0 (0)	9
	Met/SU	40 (56.3)	78 (11.2)	75 (12.8)	181 (47.5)	61 (66.3)	55 (24)
	Met/TZD	12 (16.9)	N/A	1 (0.2)	20 (5.2)	0 (0)	1 (1)
	Met/other	3 (4.2)	13 (1.9)	0	0 (0)	0 (0)	85
	SU/TZD	7 (9.9)	N/A	5 (0.9)	10 (2.6)	0 (0)	0 (0)

	SU/other	0 (0)	12(1.7)	1 (0.2)	0 (0)	0 (0)	20
	TZD/other	0 (0)	0 (0)	0 (0)	0 (0)	1 (1.1)	0 (0)
3-drugs combinations	Total	26 (8.1)	23 (3.3)	13 (2.2)	92	15	75 (9)
	Ins/SU/Met	5 (19.2)	12 (1.7)	7 (1.2)	23 (25)	4 (26.6)	6 (8)
	Ins/Met/TZD	1 (3.9)	0 (0)	0 (0)	4 (4.3)	0 (0)	0 (0)
	Ins/SU/TZD	0 (0)	0 (0)	1 (0.2)	0 (0)	0 (0)	0 (0)
	SU/Met/TZD	16 (61.5)	0 (0)	2 (0.3)	14 (15.2)	0 (0)	1 (1)
	Other	4 (15.4)	11(1.6)	3 (0.5)	51 (55.4)	11 (73.3)	65
4- drugs combinations	Total	0 (0)	0 (0)	1 (0.2)	8	1	16 (2)
	Ins/Met/SU/TZD	0 (0)	0 (0)	0 (0)	1 (12.5)	1	0 (0)
≥5-drugs combination	[any: Ins/Met/SU/TZD/other]	0 (0)	0 (0)	0 (0)	0 (0)	0 (0)	0 (0)

Numbers are N (%)
