# Supplementary material for: Association of metformin, sulfonylurea and insulin use with brain structure and function and risk of dementia and Alzheimer’s disease: Pooled analysis from 5 cohorts
Source: PLoS One. 2019 Feb 15;14(2):e0212293. doi: 10.1371/journal.pone.0212293 (PMC6377188; doi:10.1371/journal.pone.0212293)
Supplement: S3 Table — (PDF) [file pone.0212293.s003.pdf]

S3 Table. Cognitive tests used to create PC1 for global cognition in each cohort

Cohort	Tests
**FHS**	Trails making B, Hooper Visual Organization Test, Logical Memory, Visual Reproductions, Paired Associate Learning and Similarities
**SALSA**	3MSE and Word list recall
**ARIC**	Trails Making A, Trails Making B, Logical memory- immediate recall, Logical memory-delayed recall, Delayed word recall test, Animal fluency, Word fluency, Boston naming, digit symbol substitution and Digit span backwards
**RS**	Letter digit substitution, Stroop test 3, Word fluency, Word learning- delayed, Purdue pegboard test sum of right, left and both
**AGES**	California Verbal Learning test, Digit Symbol Substitution test, Figure Comparison, Stroop test Part I, Stroop test Part II, Stroop test Part III, Digit span backwards
**IDCD**	Boston naming, Diamond cancellation, Trails Making A, Trails Making B, Word-list Delayed recall, Word-list immediate recall, Similarities, Letter fluency, Animal fluency, Praxis test from ADAS cog, Logical memory delayed recall, logical memory immediate recall, Digit span backwards, Digit span forward and Digit symbol substitution
