# Supplementary material for: Association of metformin, sulfonylurea and insulin use with brain structure and function and risk of dementia and Alzheimer’s disease: Pooled analysis from 5 cohorts
Source: PLoS One. 2019 Feb 15;14(2):e0212293. doi: 10.1371/journal.pone.0212293 (PMC6377188; doi:10.1371/journal.pone.0212293)
Supplement: S4 Table — S4a Table: Baseline characteristics of FHS study participants by history of diabetes: prospective analyses of incident dementia/AD S4b Table: Baseline characteristics of FHS study participants: longitudinal analyses of change in cognition (including prevalent dementia) S4c Table: Baseline characteristics of FHS study participants by history of diabetes: cross-sectional analyses S4d Table: Baseline characteristics of AGES study participants by diabetes status: prospective analyses S4e Table: Baseline characteristics of AGES study participants: longitudinal analyses of change in cognition S4f Table: Baseline characteristics of AGES study participants by diabetes status: cross-sectional analyses S4g Table: Baseline characteristics of SALSA study participants by diabetes status: prospective analyses S4h Table: Baseline characteristics of SALSA study participants: longitudinal analyses of change in cognition S4i Table: Baseline characteristics of SALSA study participants by diabetes status: cross-sectional analyses S4j Table: Baseline characteristics of ARIC study participants by diabetes status: prospective analyses S4k Table: Baseline characteristics of ARIC study participants: longitudinal analyses of change in cognition S4l Table: Baseline characteristics of ARIC study participants by diabetes status: cross-sectional analyses S4m Table: Baseline characteristics of RS study participants by diabetes status: prospective analyses S4n Table: Baseline characteristics of RS study participants: longitudinal analyses of change in cognition S4o Table: Baseline characteristics of RS study participants by diabetes status: cross-sectional analyses S4p Table: Baseline characteristics of IDCD study participants by diabetes status: cross-sectional analyses. (PDF) [file pone.0212293.s004.pdf]

S4. Descriptives

S4a Table: Baseline characteristics of FHS study participants by history of diabetes: prospective analyses of incident dementia/AD

	Diabetes (history)		Total
	Yes	No	
N	277	1337	1614
Incident Dementia, n (%)	38 (13.72)	95 (7.11)	133 (8.24)
Incident Alzheimer's Disease, n (%)	30 (10.83)	70 (5.24)	100 (6.20)
Incident Dementia (10 yr), n (%)	35 (12.64)	81 (6.06)	116 (7.19)
Incident Alzheimer's Disease (10 yr), n (%)	27 (9.75)	60 (4.49)	87 (5.39)
Age (mean±SD)	70.08 ± 5.86	68.47 ± 5.64	68.75 ± 5.71
Women, n (%)	123 (44.40)	727 (54.38)	850 (52.66)
ApoEε4, n (%)	63 (23.16)	294 (22.29)	357 (22.44)
Duration with DM (mean±SD)	N/A	N/A	N/A
Blood glucose in fasting state (mean±SD)	150.57 ± 44.06	98.15 ± 9.66	106.79 ± 27.85
Blood glucose in random state (mean±SD)	N/A	N/A	N/A
HbA1C (mean±SD)<br>(median, (Q1, Q3))	7.21 ± 1.48<br>6.93 (6.16, 7.78)	5.52 ± 0.58<br>5.48 (5.18, 5.82)	5.80 ± 1.02<br>5.59 (5.24, 6.05)
Systolic blood pressure (mean±SD)	136.63 ± 19.64	131.43 ± 18.89	132.32 ± 19.11
Total Cholesterol (mean±SD)	187.19 ± 40.40	201.35 ± 35.32	199.02 ± 36.57
HDL (mean±SD)	45.88 ± 13.99	54.55 ± 17.14	53.13 ± 16.97
HTN (stage≥1) (JNC7 guideline), n (%)	214 (77.26)	726 (54.34)	940 (58.28)
HTN (stage≥1) (JNCI8 guideline), n (%)	N/A	N/A	N/A
Prevalent CVD, n (%)*	76 (27.44)	156 (11.67)	232 (14.37)
Prevalent stroke, n (%) **	20 (7.22)	23 (1.72)	43 (2.66)
Current smokers, n (%)	19 (6.86)	115 (8.61)	134 (8.31)
Education, n (%)			
No HS degree	35 (13.21)	66 (5.07)	101 (6.45)
HS degree	95 (35.85)	440 (33.79)	535 (34.14)
Some college	71 (26.79)	381 (29.26)	452 (28.84)
College degree	64 (24.15)	415 (31.87)	479 (30.57)

BMI (mean±SD)<br>(median, (Q1, Q3))	30.28 ± 5.35<br>29.59<br>(26.57, 33.55)	27.66 ± 4.70<br>27.08<br>(24.45, 30.19)	28.10 ± 4.91<br>27.45<br>(24.69, 30.86)
Physical activity index (mean±SD)<br>(median, (Q1, Q3))	37.66 ± 6.26<br>36.80<br>(33.20, 41.70)	38.28 ± 6.52<br>37.40<br>(33.70, 41.80)	38.18 ± 6.48<br>37.20<br>(33.60, 41.80)

S4b Table: Baseline characteristics of FHS study participants: longitudinal analyses of change in cognition (including prevalent dementia)

N	194
Baseline visit (years)	1998-2001
Duration of follow-up, years (mean±SD)	6.48±1.27
Age (mean±SD)	63.75±8.62
Women, n (%)	80 (41.24)
ApoEε4, n (%)	43 (22.75)
Duration with DM (mean±SD)	--
Blood glucose in fasting state (mean±SD)	--
Blood glucose in random state (mean±SD)	--
HbA1C (mean±SD)<br>(median, (Q1, Q3))	7.23±1.74<br>6.82 (6.07,7.90)
Systolic blood pressure (mean±SD)	131.21±16.89
Total Cholesterol (mean±SD)	192.28±38.50
HDL (mean±SD)	44.38±13.89
HTN (stage≥1) (JNC7 guideline), n (%)	133 (68.56)
HTN (stage≥1) (JNCI8 guideline), n (%)	--
Prevalent CVD, n (%)*	49 (25.26)
Prevalent stroke, n (%) **	8 (4.12)
Current smokers, n (%)	19 (9.79)
Education, n (%)	
No HS degree	12 (6.19)
HS degree	63 (32.47)
Some college	64 (32.99)
College degree	55 (28.35)
BMI (mean±SD)<br>(median, (Q1, Q3))	30.99±5.82<br>29.91 (26.89,34.41)
Physical activity index (mean±SD)<br>(median, (Q1, Q3))	37.42±6.08<br>36.80 (33.20,40.60)
Prevalent Dementia, n (%)	18 (9.28)

S4c Table: Baseline characteristics of FHS study participants by history of diabetes: cross-sectional analyses

	Cognitive data exists			MRI data exists		
	Diabetes (history)		Total	Diabetes (history)		Total
	Yes	No		Yes	No	
N	322	1699	2021	234	1399	1633
Age (mean±SD)	70.18 ± 8.78	66.55 ± 8.96	67.12 ± 9.03	69.40 ± 8.44	66.03 ± 8.86	66.51 ± 8.88
Women, n (%)	127 (39.44)	959 (56.44)	1086 (53.74)	86 (36.75)	786 (56.18)	872 (53.40)
ApoEε4, n (%)	78 (24.92)	370 (22.17)	448 (22.60)	49 (21.59)	312 (22.71)	361 (22.55)
Duration with DM (mean±SD)	N/A	N/A	N/A	N/A	N/A	N/A
Blood glucose in fasting state (mean±SD)	140.35 ± 42.29	100.06 ± 9.10	106.46 ± 23.87	140.91 ± 41.81	99.91 ± 9.07	105.80 ± 22.97
Blood glucose in random state (mean±SD)	N/A	N/A	N/A	N/A	N/A	N/A
HbA1C (mean±SD) (median, (Q1, Q3))	6.66 ± 1.15<br>6.40<br>(5.90, 7.00)	5.55 ± 0.29<br>5.50<br>(5.40, 5.70)	5.73 ± 0.66<br>5.60<br>(5.40, 5.80)	6.63 ± 1.10<br>6.40<br>(5.90, 7.00)	5.55 ± 0.28<br>5.50<br>(5.40, 5.70)	5.71 ± 0.62<br>5.60<br>(5.40, 5.80)
Systolic blood pressure (mean±SD)	132.48 ± 17.85	127.61 ± 16.79	128.38 ± 17.06	132.98 ± 18.80	127.00 ± 16.55	127.85 ± 17.01
Total Cholesterol (mean±SD)	164.79 ± 35.66	189.73 ± 36.05	185.81 ± 37.11	164.77 ± 34.70	190.55 ± 35.63	186.89 ± 36.61
HDL (mean±SD)	47.85 ± 15.23	58.76 ± 17.67	57.05 ± 17.76	48.35 ± 14.90	59.09 ± 17.76	57.56 ± 17.78
HTN (stage≥1) (JNC7 guideline), n (%)	286 (88.82)	961 (56.56)	1247 (61.70)	205 (87.61)	752 (53.75)	957 (58.60)
HTN (stage≥1) (JNCI8 guideline), n (%)	N/A	N/A	N/A	N/A	N/A	N/A
Prevalent CVD, n (%)*	73 (22.67)	155 (9.12)	228 (11.28)	48 (20.51)	107 (7.65)	155 (9.49)

Prevalent stroke, n (%) **	26 (8.07)	33 (1.94)	59 (2.92)	13 (5.56)	26 (1.86)	39 (2.39)
Dementia, n (%)	17 (5.28)	25 (1.47)	42 (2.08)	6 (2.56)	16 (1.14)	22 (1.35)
Current smokers, n (%)	20 (6.21)	140 (8.24)	160 (7.92)	15 (6.41)	112 (8.01)	127 (7.78)
Education, n (%)						
No HS degree	16 (4.97)	44 (2.59)	60 (2.97)	10 (4.27)	30 (2.14)	40 (2.45)
HS degree	112 (34.78)	419 (24.66)	531 (26.27)	85 (36.32)	336 (24.02)	421 (25.78)
Some college	100 (31.06)	520 (30.61)	620 (30.68)	62 (26.50)	423 (30.24)	485 (29.70)
College degree	94 (29.19)	716 (42.14)	810 (40.08)	77 (32.91)	610 (43.60)	687 (42.07)
BMI (mean±SD)<br>(median, (Q1, Q3))	31.15 ± 5.85<br>30.33<br>(27.17, 34.58)	27.67 ± 4.99<br>27.08<br>(24.21,30.43)	28.22 ± 5.29<br>27.69<br>(24.53,31.14)	30.62 ± 5.44<br>29.89<br>(26.83,34.24)	27.53 ± 4.89<br>26.95<br>(24.13, 30.21)	27.97 ± 5.09<br>27.41<br>(24.40,30.67)
Physical activity index<br>(mean±SD)<br>(median, (Q1, Q3))	33.50 ± 4.53<br>33.10<br>(30.60,36.20)	35.35 ± 5.70<br>34.60<br>(31.60,38.00)	35.06 ± 5.57<br>34.30<br>(31.50,37.60)	33.76 ± 4.23<br>33.50<br>(31.00,36.30)	35.54 ± 5.84<br>34.80<br>(31.80, 36.30)	35.29 ± 5.67<br>34.50<br>(31.60,38.00)

S4d Table: Baseline characteristics of AGES study participants by diabetes status: prospective analyses

	Diabetes		Total
	Yes	No	
N	623	4,494	5,117
Age (mean±SD)	76.4±5.3	76.3±5.5	76.3±5.5
N (%) women	285(45.8%)	2,669(59.4%)	2,954(57.7%)
N (%) with ApoEε4	154(25.0%)	1,248(27.8%)	1,402(27.5%)
Duration with DM (mean±SD) (median(Q1-Q3)); yrs	12.5±11.6<br>9.5 (3.0-19.0)	N/A	*Available on a subsample*
Blood glucose in fasting state (mean±SD); (median(Q1-Q3); mmol/L	7.85±2.15<br>7.4 (6.6-8.5)	5.50±0.51	5.78±1.17<br>5.5 (5.2-6.0)
Blood glucose in random state (mean±SD)	N/A	N/A	N/A
HbA1C (mean±SD) %	6.44±0.89<br>6.2 (5.90-6.70)	5.59±0.33	5.69±0.52<br>5.60 (5.40-5.90)
Systolic blood pressure (mean±SD), mmHg	145.7±20.8	141.9±20.3	142.3±20.4
Total Chol (mean±SD), mmol/L	5.16±1.15	5.70±1.15	5.63±1.17
HDL (mean±SD), mmol/L	1.38±0.39	1.62±0.45	1.59±0.45
N (%) with HTN (stage≥1)	566(90.9%)	3,555(79.1%)	4,121(80.5%)
N (%) with prevalent CVD	191(30.9%)	931(20.9%)	1,122(22.1%)
N (%) with prevalent stroke	51(8.2%)	203(4.6%)	254(5.0%)
N (%) current smokers	74(12.0%)	546(12.3%)	620 (12.2%)
Years of education (mean±SD)			
**Education categories**			
Primary education	118(20.2%)	994(22.7%)	1,112(22.4%)
Secondary	297(50.9%)	2190(50.0%)	2,487(50.1%)
College	87(14.9%)	700(16.0%)	787(15.9%)
University	82(14.0%)	498(11.4%)	580(11.7%)
BMI (mean±SD), kg/m^2	28.91±4.80	26.84±4.32	27.10±4.43

Physical activity index (mean±SD)			
**<u>Physical activity in current life</u>**			
Never/rarely/occasionally	421(72.7%)	2,961(68.0%)	3,382 (68.5%)
Moderate/high	158(27.3%)	1,394(32.0%)	1,552(31.5%)

S4e Table: Baseline characteristics of AGES study participants: longitudinal analyses of change in cognition

N	287
Baseline visit (years)	2002-2006
Duration of follow-up(mean±SD)	5.1±0.2
Age (mean±SD)	74.5±4.3
Women, n (%)	134(46.7%)
ApoEε4, n (%)	78(27.4%)
Duration with DM (mean±SD)	-
Blood glucose in fasting state (mean±SD)mmol/L	7.8±2.0
Blood glucose in random state (mean±SD)	-
HbA1C (mean±SD)<br>(median, (Q1, Q3))	-
Systolic blood pressure (mean±SD)	145.4±20.1
Total Cholesterol (mean±SD)	5.1±1.2
HDL (mean±SD)	-
HTN (stage≥1) (JNC7 guideline), n (%)	258(89.9%)
HTN (stage≥1) (JNCI8 guideline), n (%)	-
Prevalent CVD, n (%)*	79(27.7%)
Prevalent stroke, n (%) **	13(4.5%)
Current smokers, n (%)	27(9.4%)
Education, n (%)	
No HS degree	48(16.8%)
HS degree	155(54.2%)
Some college	40(14.0%)
College degree	43(15.0%)
BMI (mean±SD)<br>(median, (Q1, Q3))	29.3±4.6<br>28.9(26.2-32.1)
Physical activity index (mean±SD)<br>(median, (Q1, Q3)) present moderate/high physical activity, n (%)	94(33.1%)

Prevalent Dementia, n (%)	6 (2.1%)

S4f Table: Baseline characteristics of AGES study participants by diabetes status: cross-sectional analyses

	Cognitive data exists			MRI data exists		
	Diabetes			Diabetes		
	Yes	No	Total	Yes	No	Total
N	694	4,816	5,510	N/A	N/A	N/A
Age (mean±SD), yrs	77.0±5.6	76.7±5.7	76.7±5.7	N/A	N/A	N/A
N (%) women	312(45.0%)	2,854(59.3%)	3,166(57.5%)	N/A	N/A	N/A
N (%) with ApoEε4	173(25.2%)	1384(28.8%)	1,557(28.4%)	N/A	N/A	N/A
Duration with DM (mean±SD), yrs	12.4±11.5<br>9.0(3.0-19.0)[a]	-	Available on a subsample	N/A	N/A	N/A
Blood glucose in fasting state (mean±SD) mmol/L	7.82±2.12<br>7.4(6.6-8.4)	5.50±0.51	5.79±1.18<br>5.5(5.2-6.0)	N/A	N/A	N/A
Blood glucose in random state (mean±SD)	N/A	N/A	N/A	N/A	N/A	N/A
HbA1C (mean±SD), %	6.44±0.90<br>6.2(5.9-6.8)	5.59±0.33	5.69±0.52<br>5.6(5.4-5.9)	N/A	N/A	N/A
Systolic blood pressure (mean±SD)	144.9±21.0	141.9±20.5	142.2±20.5	N/A	N/A	N/A
Total Chol (mean±SD),mmol/L	5.19±1.16	5.70±1.15	5.63±1.16	N/A	N/A	N/A
HDL (mean±SD),mmol/L	1.38±0.39	1.62±0.45	1.59±0.45	N/A	N/A	N/A
N (%) with HTN (stage≥1)	628(90.5%)	3820(79.3%)	4,448(80.7%)	N/A	N/A	N/A
N (%) with prevalent CVD	221(32.1%)	1017(21.3%)	1,238(22.7%)	N/A	N/A	N/A
N (%) with prevalent stroke	62(9%)	241(5.1%)	303(5.6%)	N/A	N/A	N/A
N (%) with dementia	N/A	N/A	N/A	N/A	N/A	N/A
N (%) current smokers	80(11.7%)	586(12.3%)	666(12.2%)	N/A	N/A	N/A

**Education categories**				N/A	N/A	N/A
Primary education	133(21.2%)	1113(23.9%)	1246(23.6%)			
Secondary	322(51.3%)	2310(49.6%)	2632(49.8%)			
College	90(14.3%)	721(15.5%)	811(15.4%)			
University	83(13.2%)	512(11.0%)	595(11.3%)			
BMI (mean±SD), kg/m^2	28.85±4.79	26.78±4.32	27.04±4.43	N/A	N/A	N/A
Physical activity index (mean±SD)				N/A	N/A	N/A
**Physical activity in current life**						
Never/rarely/occasionally						
Moderate/high						
	460(73.8%)	3190(68.9%)	3650(69.5%)			
	163(26.2%)	1440(31.1%)	1603(30.5%)			

[a] Median(Q1-Q3) was also presented for continuous data with skewed distribution

S4g Table: Baseline characteristics of SALSA study participants by diabetes status: prospective analyses

	Diabetes		Total
	Yes	No	
N	555	1156	1711
Age (mean±SD)	69.9±6.6	70.5±7.0	70.3±6.8
N (%) women	313 (56.4)	687 (59.4)	1003 (58.3)
N (%) with ApoEε4	56 (11.1)	158 (15.1)	215 (13.8)
Duration with DM (mean±SD)	12.9±11.3	-	12.9±11.3
Blood glucose in fasting state (mean±SD)	157.8±58.3	93.8±12.0	115.0±46.1
Blood glucose in random state (mean±SD)	-	-	-
HbA1C (mean±SD)	-	-	-
Systolic blood pressure (mean±SD)	140.4±19.7	137.4±19.0	138.4±19.3
Total Chol (mean±SD)	208.1±42.8	214.6±38.1	212.5±39.8
HDL (mean±SD)	48.2±11.8	53.6±14.0	51.8±13.5
N (%) with HTN (stage≥1)	415 (74.8)	635 (54.9)	1050 (61.1)
N (%) with prevalent CVD	269 (48.5)	352 (30.5)	621 (36.3)
N (%) with prevalent stroke	71 (12.8)	74 (6.4)	145 (8.5)
N (%) current smokers	54 (9.7)	141 (12.2)	195 (11.4)
Years of education (mean±SD)	7.2±5.4	7.3±5.3	7.3±5.3
BMI (mean±SD)	31.0±6.0	29.1±5.4	29.7±5.7
Physical activity index (mean±SD)	16.3±5.4	17.6±5.4	17.2±5.5

S4h Table: Baseline characteristics of SALSA study participants: longitudinal analyses of change in cognition

N	586
Age (mean±SD)	70.3±6.9
N (%) women	332 (56.7)
N (%) with ApoEε4	60 (11.2)
Duration with DM (mean±SD)	12.8±11.3
Blood glucose in fasting state (mean±SD)	157.0±58.3
Blood glucose in random state (mean±SD)	-
HbA1C (mean±SD)	-
Systolic blood pressure (mean±SD)	140.3±20.0
Total Chol (mean±SD)	207.5±42.7
HDL (mean±SD)	48.3±11.8
N (%) with HTN (stage≥1)	439 (74.9)
N (%) with prevalent CVD	288 (49.2)
N (%) with prevalent stroke	87 (14.9)
N (%) with dementia	31 (5.3)
N (%) current smokers	57 (9.7)
Years of education (mean±SD)	7.1±5.4
BMI (mean±SD)	30.9±6.0
Physical activity index (mean±SD)	16.3±5.4
Prevalent dementia, n(%)	31 (5.3)

S4i Table: Baseline characteristics of SALSA study participants by diabetes status: cross-sectional analyses

	Cognitive data exists			MRI data exists		
	Diabetes		Total	Diabetes		Total
	Yes	No		Yes	No	
N	586	1192	1778	85	121	206
Age (mean±SD)	70.3±6.9	70.8±7.2	70.7±7.1	70.5±6.9	71.2±6.5	70.9±6.7
N (%) women	332 (56.7)	708 (59.4)	1040 (58.5)	46 (54.1)	69 (57.0)	115 (55.8)
N (%) with ApoEε4	60 (11.2)	164 (15.2)	224 (13.9)	17 (20.0)	29 (24.2)	46 (22.4)
Duration with DM (mean±SD)	12.8±11.3	-	12.8±11.3	11.5±13.4	-	11.5±13.4
Blood glucose in fasting state (mean±SD)	157.0±58.3	93.7±12.0	115.0±46.2	156.0±50.2	93.6±10.5	119.7±45.4
Blood glucose in random state (mean±SD)	-	-	-	-	-	-
HbA1C (mean±SD)	-	-	-	-	-	-
Systolic blood pressure (mean±SD)	140.3±20.0	137.4±19.1	138.4±19.4	140.9±19.8	137.7±19.3	139.0±19.5
Total Chol (mean±SD)	207.5±42.7	214.2±38.4	211.9±40.0	200.2±36.3	213.1±39.4	207.7±38.6
HDL (mean±SD)	48.3±11.8	53.6±14.0	51.8±13.6	48.4±12.2	54.2±13.6	51.8±13.3
N (%) with HTN (stage≥1)	439 (74.9)	661 (55.5)	1100 (61.9)	64 (75.3)	71 (58.7)	135 (65.5)
N (%) with prevalent CVD	288 (49.2)	371 (31.1)	659 (37.1)	36 (42.4)	43 (35.5)	79 (38.4)
N (%) with prevalent stroke	87 (14.9)	81 (6.8)	168 (9.5)	14 (16.5)	13 (10.7)	27 (13.1)
N (%) with dementia	31 (5.3)	37 (3.1)	68 (3.8)	12 (14.1)	10 (8.3)	22 (10.7)
N (%) current smokers	57 (9.7)	146 (12.3)	203 (11.4)	11 (12.9)	10 (8.3)	21 (10.2)
Years of education (mean±SD)	7.1±5.4	7.3±5.3	7.2±5.3	7.7±5.4	8.1±5.6	8.0±5.5
BMI (mean±SD)	30.9±6.0	29.1±5.4	29.7±5.6	30.6±4.9	29.2±4.9	29.8±5.0
Physical activity index (mean±SD)	16.3±5.4	17.6±5.4	17.2±5.5	15.6±4.9	17.3±5.5	16.6±5.3

S4j Table: Baseline characteristics of ARIC study participants by diabetes status: prospective analyses

	Diabetes		Total
	Yes	No	
N	1197	9544	10741
Age (mean±SD)	64.05±5.75	63.25±5.65	63.34±5.66
N (%) women	646(53.97)	5331(55.86)	5977(55.65)
N (%) with ApoE ε4	339(28.32)	2798(29.32)	3137(29.21)
Duration with DM (mean±SD)	5.79±3.48	-	-
Blood glucose in fasting state (mean±SD)	164.12±60.39	102.47±19.53	108.04±31.44
Blood glucose in random state (mean±SD)	209.91±93.03	107.19±30.07	171.08±90.57
HbA1C (mean±SD)	-	-	-
Systolic blood pressure (mean±SD)	132.73±19.96	126.66±18.61	127.33±18.86
Total Chol (mean±SD)	196.96±39.58	201.36±36.67	200.87±37.03
HDL (mean±SD)	44.51±14.05	50.57±16.56	49.90±16.41
N (%) with HTN (stage≥1)	805(67.25)	4229(44.31)	5034(46.87)
N (%) with prevalent CVD	219(18.30)	692(7.25)	911(8.48)
N (%) with prevalent stroke	55(4.59)	176(1.84)	231(2.15)
N (%) current smokers	160(13.37)	1399(14.66)	1559(14.51)
Years of education (mean±SD)	13.46±4.47	14.77±4.28	14.62±4.32
BMI (mean±SD)	31.73±5.99	28.40±5.39	28.77±5.56
Physical activity index (mean±SD)	2.38±0.76	2.57±0.82	2.55±0.81

S4k Table: Baseline characteristics of ARIC study participants: longitudinal analyses of change in cognition

N	1,197
Baseline visit (years)	1996-1998
Duration of follow-up(mean±SD)	11.81±4.63
Age (mean±SD)	64.05±5.75
Women, n (%)	646(53.97)
ApoEε4, n (%)	339(28.32)
Duration with DM (mean±SD)	5.79±3.48
Blood glucose in fasting state (mean±SD)mmol/L	164.12±60.39
Blood glucose in random state (mean±SD)	209.91±93.03
HbA1C (mean±SD) (median, (Q1, Q3))	-
Systolic blood pressure (mean±SD)	132.73±19.96
Total Cholesterol (mean±SD)	196.96±39.58
HDL (mean±SD)	44.51±14.05
HTN (stage≥1), n (%)	805(67.25)
Prevalent CVD, n (%)*	219(18.30)
Prevalent stroke, n (%) **	55(4.59)
Current smokers, n (%)	160(13.37)
Education, years	13.46±4.47
BMI (mean±SD) (median, (Q1, Q3))	31.73±5.99
Physical activity index (mean±SD) (median, (Q1, Q3)) present moderate/high physical activity, n (%)	2.38±0.76
Prevalent Dementia, n (%)	0 (0)

S4I Table: Baseline characteristics of ARIC study participants by diabetes status: cross-sectional analyses

	Cognitive data exists			MRI data exists		
	Diabetes		Total	Diabetes		Total
	Yes	No		Yes	No	
N	1732	3724	5456	575	1177	1752
Age (mean±SD)	75.77±5.16	75.68±5.24	75.71±5.21	75.76±5.18	75.66±5.23	75.69±5.21
N (%) women	981(56.64)	2195(58.94)	3176(58.21)	340(59.13)	702(59.64)	1042(59.47)
N (%) with ApoEε4	498(28.75)	1073(28.81)	1571(28.79)	160(27.83)	348(29.57)	508(29.00)
Duration with DM (mean±SD)	9.92±6.60	-	9.92±6.60	9.85±6.55	-	9.85±6.55
Blood glucose in fasting state (mean±SD)	129.60±38.14	104.26±13.90	112.08±26.81	129.48±38.29	104.19±13.76	111.95±26.76
Blood glucose in random state (mean±SD)	145.77±56.21	105.33±16.39	120.38±41.30	145.77±56.21	105.33±16.39	120.38±41.30
HbA1C (mean±SD)	6.58±1.10	5.64±0.40	5.93±0.82	6.56±1.09	5.64±0.39	5.92±0.81
Systolic blood pressure (mean±SD)	129.92±17.33	129.73±18.00	129.79±17.79	129.86±17.29	129.64±17.96	129.71±17.75
Total Chol (mean±SD)	172.03±40.46	191.33±42.08	185.35±42.52	172.21±40.61	191.27±42.12	185.40±42.57
HDL (mean±SD)	49.40±12.77	55.29±14.34	53.46±14.14	49.43±12.80	55.27±14.33	53.47±14.14
N (%) with HTN (stage≥1)	1475(85.16)	2582(69.33)	4057(74.36)	485(84.35)	834(70.86)	1319(75.29)
N (%) with prevalent CVD	328(18.94)	463(12.43)	791(14.50)	63(10.96)	100(8.50)	163(9.30)
N (%) with prevalent stroke	87(5.02)	105(2.82)	192(3.52)	25(4.35)	36(3.06)	61(3.48)
N (%) with dementia	76(4.40)	112(3.01)	188(3.45)	34(5.91)	57(4.84)	91(5.19)
N (%) current smokers	96(5.54)	219(5.88)	315(5.77)	25(4.35)	63(5.35)	88(5.02)
Years of education (mean±SD)	14.69±4.13	15.92±4.18	15.54±4.20	14.71±4.13	15.91±4.18	15.54±4.20
BMI (mean±SD)	30.43±5.71	27.52±5.25	28.42±5.56	30.41±5.74	27.52±5.24	28.41±5.56
Physical activity index (mean±SD)	2.15±0.60	2.36±0.63	2.30±0.63	2.15±0.60	2.36±0.64	2.30±0.63

S4m Table: Baseline characteristics of RS study participants by diabetes status: prospective analyses

	Diabetes		Total
	Yes	No	
N	608	5149	5757
Age (mean±SD)	63.6 (7.8)	60.9 (8.8)	61.2 (8.9)
N (%) women	274 (45.1)	2969 (57.7)	3243 (56.3)
N (%) with ApoEε4	158 (27.7)	1408 (28.6)	1566 (28.7)
Duration with DM (mean±SD)	NA	-	NA
Blood glucose in fasting state (mean±SD)	7.9 (2.4)	5.3 (0.6)	5.6 (1.2)
Blood glucose in random state (mean±SD)	NA	NA	NA
HbA1C (mean±SD)	NA	NA	NA
Systolic blood pressure (mean±SD)	144.9 (20.9)	136.5 (20.1)	137.4 (20.3)
Total Chol (mean±SD)	5.1 (1.2)	5.6 (1.0)	5.6 (1.0)
HDL (mean±SD)	1.2 (0.4)	1.4 (0.4)	1.4 (0.4)
N (%) with HTN (stage≥1)	391 (64.3)	2454 (47.7)	2845 (49.5)
N (%) with prevalent CVD	77 (12.7)	261 (5.1)	338 (5.9)
N (%) with prevalent stroke	35 (5.8)	133 (2.6)	168 (3.0)
N (%) current smokers	139 (22.9)	1190 (23.1)	1329 (23.1)
Years of education			
Low (%)	215 (35.4)	1473 (28.6)	1688 (29.3)
Middle (%)	273 (44.9)	2425 (47.1)	2698 (46.9)
High (%)	112 (18.4)	1196 (23.2)	1308 (22.7)
BMI (mean±SD)	30.5 (5.0)	27.4 (4.2)	27.7 (4.4)
Physical activity index (mean±SD)	55.8 (78.4)	59.9 (58.3)	59.6 (60.4)

S4n Table: Baseline characteristics of RS study participants: longitudinal analyses of change in cognition

N	250
Baseline visit (years)	2004-2008
Duration of follow-up	5.8±0.5
Age (mean±SD)	60.7±6.8
Women, n (%)	103 (41.2)
ApoEε4, n (%)	63 (25.2)
Duration with DM (mean±SD)	NA
Blood glucose in fasting state (mean±SD)	8.0±2.8
Blood glucose in random state (mean±SD)	NA
HbA1C (mean±SD)<br>(median, (Q1, Q3))	NA
Systolic blood pressure (mean±SD)	141.6±19.4
Total Cholesterol (mean±SD)	5.0±1.2
HDL (mean±SD)	1.2±0.3
HTN (stage≥1) (JNC7 guideline), n (%)	180 (72.0)
HTN (stage≥1) (JNCI8 guideline), n (%)	152 (60.8)
Prevalent CVD, n (%)*	30 (12.0)
Prevalent stroke, n (%) **	7 (2.8)
Current smokers, n (%)	63 (25.2)
Years of education, n (%)	
Low	66 (26.4)
Middle	130 (520)
High	54 (21.6)
BMI (mean±SD)<br>(median, (Q1, Q3))	30.8±4.9<br>30.0(27.2-33.2)
Physical activity index (mean±SD)<br>(median, (Q1, Q3))	66.6±81.5<br>56.7(21.3-87.9)
Prevalent Dementia, n (%)	0 (0)

S4o Table: Baseline characteristics of RS study participants by diabetes status: cross-sectional analyses

	Cognitive data exists			MRI data exists		
	Diabetes		Total	Diabetes		Total
	Yes	No		Yes	No	
N	451	4293	4744	349	3409	3758
Age (mean±SD)	62.5 (8.1)	60.3 (8.4)	60.5 (8.4)	60.9 (7.6)	58.7 (7.5)	58.9 (7.5)
N (%) women	199 (44.1)	2493 (58.1)	2692 (56.7)	144 (41.3)	1890 (55.4)	2034 (54.1)
N (%) with ApoEε4	119 (28.1)	1192 (29.1)	1311 (29.0)	93 (28.8)	954 (29.5)	1047 (29.5)
Duration with DM (mean±SD)	NA	-	NA	NA	-	NA
Blood glucose in fasting state (mean±SD)	7.8 (2.4)	5.3 (0.6)	5.6 (1.2)	7.9 (2.5)	5.3 (0.6)	5.5 (1.2)
Blood glucose in random state (mean±SD)	NA	NA	NA	NA	NA	NA
HbA1C (mean±SD)	NA	NA	NA	NA	NA	NA
Systolic blood pressure (mean±SD)	144.4 (20.3)	136.3 (19.9)	137.1 (20.2)	141.6 (19.4)	134.0 (19.1)	134.7 (1.2)
Total Chol (mean±SD)	5.1 (1.2)	5.7 (1.0)	5.6 (1.0)	5.0 (1.2)	5.6 (1.0)	5.6 (1.0)
HDL (mean±SD)	1.2 (0.4)	1.5 (0.4)	1.4 (0.4)	1.2 (0.3)	1.4 (0.4)	1.4 (0.4)
N (%) with HTN (stage≥1)	291 (64.5)	2026 (47.2)	2317 (48.8)	231 (66.2)	1548 (45.4)	1779 (47.3)
N (%) with prevalent CVD	55 (12.2)	194 (4.5)	249 (5.2)	37 (10.6)	122 (3.6)	159 (4.2)
N (%) with prevalent stroke	21 (4.7)	82 (1.9)	103 (2.2)	14 (4.0)	76 (2.2)	90 (2.4)
N (%) with dementia	0	8 (0.2)	8 (0.2)	5 (1.4)	6 (0.2)	11 (0.3)
N (%) current smokers	111 (24.6)	968 (22.5)	1079 (22.7)	97 (27.8)	809 (23.7)	906 (24.1)
Years of education						
Low (%)	145 (32.2)	1176 (27.4)	1321 (27.8)	113 (32.4)	890 (26.1)	1003 (26.7)
Middle (%)	217 (48.1)	2052 (47.8)	2269 (47.8)	155 (44.4)	1609 (47.2)	1764 (46.9)
High (%)	81 (18.0)	1024 (23.9)	1105 (23.3)	78 (22.3)	879 (25.8)	957 (25.5)
BMI (mean±SD)	30.5 (4.9)	27.4 (4.2)	27.7 (4.4)	30.2 (4.7)	27.2 (4.0)	27.5 (4.2)
Physical activity index (mean±SD)	60.0 (57.8)	54.2 (80.0)	59.7 (60.7)	52.3 (52.9)	60.6 (59.6)	59.9 (59.1)
Depression	44 (9.8)	375 (8.7)	419 (8.8)	35 (10.0)	279 (8.2)	314 (8.4)

S4p Table: Baseline characteristics of IDCD study participants by diabetes status: cross-sectional analyses

	Cognitive data exists			MRI data exists		
	Diabetes		Total	Diabetes		Total
	Yes	No		Yes	No	
N	912	N/A	N/A	125	N/A	N/A
Age (mean±SD)	72.9±4.7	N/A	N/A	71.5 (3.8)	N/A	N/A
N (%) women	539 (59%)	N/A	N/A	48 (38%)	N/A	N/A
N (%) with ApoEε4	294 Missing<br>88 (14%)	N/A	N/A	28 (22%)	N/A	N/A
Duration with DM (mean±SD)	8.7 (2.6)	N/A	N/A	8.6 (2.4)	N/A	N/A
Blood glucose in fasting state (mean±SD)	136.0±21.6	N/A	N/A	136.5 (21.4)	N/A	N/A
Blood glucose in random state (mean±SD)		N/A	N/A		N/A	N/A
Systolic blood pressure (mean±SD)	164.8±9.4	N/A	N/A	166.4 (10.1)	N/A	N/A
Total Chol (mean±SD)	180.6±24.9	N/A	N/A	179.6 (23.3)	N/A	N/A
HDL (mean±SD)	47.7±10.8	N/A	N/A	47.5 (10.7)	N/A	N/A
N (%) with HTN (stage≥1)	785 (86%)	N/A	N/A	106 (84.8)	N/A	N/A
N (%) with HTN (stage≥1)	N/A	N/A	N/A	N/A	N/A	N/A
N (%) with prevalent CVD	218 (25%) with CVD	N/A	N/A	33 (26)	N/A	N/A
N (%) with prevalent stroke	0	N/A	N/A	0	N/A	N/A
N (%) current smokers	89 (9.9%)	N/A	N/A	14 (6.4%)	N/A	N/A
Years of education (mean±SD)	13.1±3.5	N/A	N/A	13.9 (3.6)	N/A	N/A
BMI (mean±SD)	28.6±7.8	N/A	N/A	28.4 (4.5)	N/A	N/A
Physical activity index, n(%)	High 62.9%<br>Middle 31.2%<br>Low 5.8%	N/A	N/A	High 62.5%<br>Middle 29.6%<br>Low 7.9%	N/A	N/A
Depression	87 (10%)	N/A	N/A	14 (6.5%)	N/A	N/A
