# Supplementary material for: Association of metformin, sulfonylurea and insulin use with brain structure and function and risk of dementia and Alzheimer’s disease: Pooled analysis from 5 cohorts
Source: PLoS One. 2019 Feb 15;14(2):e0212293. doi: 10.1371/journal.pone.0212293 (PMC6377188; doi:10.1371/journal.pone.0212293)
Supplement: S5 Table — S5a Table: Heterogeneity statistics for the associations of diabetes drug classes with incident dementia and AD among individuals with diabetes S5b Table: Heterogeneity statistics for the associations of diabetes drug classes with cognitive performance among individuals with diabetes S5c Table: Heterogeneity statistics for the associations of diabetes drug classes with cognitive change among individuals with diabetes S5d Table: Heterogeneity statistics for the associations of diabetes drug classes with brain MRI measures among individuals with diabetes. (PDF) [file pone.0212293.s005.pdf]

S5. Assessment of Heterogeneity

S5a Table: Heterogeneity statistics for the associations of diabetes drug classes with incident dementia and AD among individuals with diabetes

		Metformin		Sulfonylurea		Insulin	
**Outcome**	**# cohorts**	**I-squared**	**95% CI**	**I-squared**	**95% CI**	**I-squared**	**95% CI**
Incident AD	4	0.54	0.00, 0.85	0.00	0.00, 0.78	0.00	0.00, 0.82
Incident Dementia	5	0.11	0.00 ,0.82	0.00	0.00, 0.74	0.00	0.00, 0.00

Adjusted for age, sex, education, physical activity, hypertension, CVD, stroke, total cholesterol, smoking, depression, BMI, HbA1C/ fasting blood glucose /random state blood glucose and ApoE4

S5b Table: Heterogeneity statistics for the associations of diabetes drug classes with cognitive performance among individuals with diabetes

		Metformin		Sulfonylurea		Insulin	
**Outcome**	**# cohorts**	**I-squared**	**95% CI**	**I-squared**	**95% CI**	**I-squared**	**95% CI**
Executive function (trails B-A)	3	0.60	0.00, 0.89	0.00	0.00, 0.86	0.43	0.00, 0.83
Word list - delayed	5	0.00	0.00, 0.73	0.31	0.00, 0.73	0.24	0.00, 0.69
Word list - combined	4	0.30	0.00, 0.75	0.30	0.00, 0.75	0.00	0.00, 0.00
Paragraph recall - delayed	3	0.00	0.00, 0.57	0.65	0.00, 0.90	0.00	0.00, 0.48
Paragraph recall - combined	3	0.00	0.00, 0.00	**0.75**	**0.15, 0.92**	0.00	0.00 ,0.73

Adjusted for age, sex, education, interval between exam cycle and cognitive assessment, Physical activity, hypertension, CVD, stroke, total cholesterol, smoking, depression, BMI, HbA1C/ fasting blood glucose /random state blood glucose and ApoE4

S5c Table: Heterogeneity statistics for the associations of diabetes drug classes with cognitive change among individuals with diabetes

		Metformin		Sulfonylurea		Insulin	
**Outcome**	**#cohorts**	**I-squared**	**95% CI**	**I-squared**	**95% CI**	**I-squared**	**95% CI**
Including Dementia Cases	5	0.56	0.00, 0.85	0.00	0.00, 0.77	0.00	0.00, 0.51
Excluding Dementia Cases	5	0.57	0.00, 0.86	0.20	0.00, 0.66	0.00	0.00, 0.51

Adjusted for age, sex, education, physical activity, hypertension, CVD, stroke, total cholesterol, smoking, depression, BMI, HbA1C/ fasting blood glucose /random state blood glucose and ApoE4

S5d Table: Heterogeneity statistics for the associations of diabetes drug classes with brain MRI measures among individuals with diabetes

		Metformin		Sulfonylurea		Insulin	
**Outcome**	**#cohorts**	**I-squared**	**95% CI**	**I-squared**	**95% CI**	**I-squared**	**95% CI**
TCBV	5	0.00	0.00, 0.66	0.22	0.00, 0.67	0.72	0.30, 0.89
HPV	5	0.00	0.00, 0.65	0.00	0.00, 0.59	0.00	0.00, 0.66
WMHV	6	0.00	0.00, 0.62	0.00	0.00, 0.57	0.01	0.00, 0.75

TCBV=Total cerebral brain volume; HPV=Hippocampal volume; WMHV=White matter hyperintensity volume

Adjusted for age and sex, interval between exam cycle and date of MRI, physical activity, hypertension, CVD, stroke, total cholesterol, smoking, depression, BMI, HbA1C/FBG/random blood glucose and ApoE4
