# Supplementary material for: Association of metformin, sulfonylurea and insulin use with brain structure and function and risk of dementia and Alzheimer’s disease: Pooled analysis from 5 cohorts
Source: PLoS One. 2019 Feb 15;14(2):e0212293. doi: 10.1371/journal.pone.0212293 (PMC6377188; doi:10.1371/journal.pone.0212293)
Supplement: S6 Table — S6a Table: Associations of diabetes drug classes with risk of dementia/AD among individuals with diabetes who receive diabetes medications S6b Table: Associations of diabetes drug classes with cognitive performance among individuals with diabetes who receive diabetes medications S6c Table: Associations of diabetes drug classes with change in global cognition among individuals with diabetes who receive diabetes medications S6d Table: Associations of diabetes drug classes with brain MRI measures among individuals with diabetes who receive diabetes medications. (PDF) [file pone.0212293.s006.pdf]

S6. Analysis among a subsample of participants with diabetes who take diabetes medications (excluding those who are on life-style change only)

S6a Table: Associations of diabetes drug classes with risk of dementia/AD among individuals with diabetes who receive diabetes medications

			Metformin		Sulfonylurea		Insulin	
	**Outcome**	**# cohorts**	**HR (95% CI)**	**p-value**	**HR (95% CI)**	**p-value**	**HR (95% CI)**	**p-value**
Model 1	Incident AD	4	1.4 (0.8, 2.46)	0.235	0.91 (0.52, 1.57)	0.726	1.52 (0.81, 2.84)	0.195
	Incident Dementia	5	1.15 (0.84, 1.59)	0.381	0.73 (0.55, 0.96)	**0.025**	1.41 (1.05, 1.9)	**0.021**
Model 2	Incident AD	4	1.87 (0.96, 3.66)	0.067	0.89 (0.44, 1.78)	0.735	1.43 (0.6, 3.43)	0.421
	Incident Dementia	5	1.27 (0.89, 1.79)	0.186	0.69 (0.52, 0.93)	**0.015**	1.50 (1.09, 2.06)	**0.014**
Model 3	Incident AD	4	1.75 (0.87, 3.55)	0.119	0.88 (0.44, 1.78)	0.722	1.39 (0.54, 3.53)	0.494
	Incident Dementia	5	1.28 (0.89, 1.84)	0.187	0.67 (0.5, 0.91)	**0.010**	1.55 (1.12, 2.15)	**0.009**
Model 4	Incident AD	4	1.48 (0.59, 3.72)	0.405	0.46 (0.16, 1.31)	0.148	1.07 (0.36, 3.15)	0.907
	Incident Dementia	5	1.31 (0.88, 1.94)	0.182	0.64 (0.46, 0.88)	**0.007**	1.49 (1.07, 2.07)	**0.018**

Model 1 is adjusted for age, sex and education
Model 2 is additionally adjusted for Physical activity, hypertension, CVD, stroke, total cholesterol, smoking, depression and BMI
Model 3 is additionally adjusted for HbA1C/ fasting blood glucose /random state blood glucose, ApoE4
Model 4 is additionally adjusted for eGFR

S6b Table: Associations of diabetes drug classes with cognitive performance among individuals with diabetes who receive diabetes medications

			Metformin			Sulfonylurea			Insulin		
	**Outcome**	**# cohorts**	**Estimate**	**SE**	**p-value**	**Estimate**	**SE**	**p-value**	**Estimate**	**SE**	**p-value**
Model 1	Global cognition	6	0.081	0.034	**0.016**	-0.037	0.034	0.283	-0.146	0.039	**<0.001**
	Executive function (trails B-A)	3	0.067	0.050	0.179	-0.017	0.049	0.736	-0.036	0.058	0.535
	Executive function (digit span backwards)	2	-0.091	0.076	0.231	-0.108	0.073	0.142	-0.036	0.103	0.725
	Word list - delayed	5	0.027	0.037	0.462	-0.011	0.037	0.764	-0.070	0.043	0.107
	Word list - combined	4	-0.056	0.051	0.274	-0.018	0.010	0.071	0.024	0.063	0.700
	Paragraph recall - delayed	3	0.119	0.045	**0.008**	0.014	0.044	0.740	-0.065	0.051	0.202
	Paragraph recall - combined	3	0.111	0.045	**0.013**	0.017	0.044	0.696	-0.077	0.051	0.129
Model 2	Global cognition	6	0.026	0.034	0.453	-0.053	0.034	0.126	-0.071	0.040	0.076
	Executive function (trails B-A)	3	0.050	0.052	0.329	-0.014	0.051	0.787	-0.017	0.061	0.777
	Executive function (digit span backwards)	2	-0.117	0.077	0.131	-0.094	0.075	0.208	0.008	0.109	0.940
	Word list - delayed	5	-0.010	0.039	0.796	-0.018	0.038	0.643	-0.037	0.045	0.414
	Word list - combined	4	-0.074	0.051	0.146	0.002	0.010	0.855	0.077	0.062	0.217
	Paragraph recall - delayed	3	0.085	0.048	0.077	-0.018	0.046	0.692	-0.010	0.055	0.856
	Paragraph recall - combined	3	0.070	0.048	0.142	-0.015	0.046	0.749	-0.017	0.055	0.751
Model 3	Global cognition	6	0.015	0.035	0.666	-0.040	0.035	0.246	-0.026	0.042	0.531
	Executive function (trails B-A)	3	0.051	0.052	0.330	-0.001	0.052	0.978	0.032	0.066	0.632
	Executive function (digit span backwards)	2	-0.143	0.078	0.065	-0.080	0.075	0.285	0.045	0.109	0.678
	Word list - delayed	5	-0.029	0.039	0.463	-0.016	0.039	0.683	0.000	0.048	0.995
	Word list - combined	4	-0.084	0.052	0.106	-0.023	0.053	0.666	0.088	0.064	0.170
	Paragraph recall - delayed	3	0.067	0.048	0.165	-0.022	0.048	0.645	0.017	0.060	0.780
	Paragraph recall - combined	3	0.047	0.048	0.331	-0.017	0.048	0.724	0.011	0.059	0.856

Model 1: Age, sex, education and interval between exam cycle and the cognitive assessment

Model 2: Model 1 + Physical activity, hypertension, CVD, stroke, total cholesterol, smoking, depression, BMI

Model 3: Model 2+HbA1C/ fasting blood glucose /random state blood glucose and ApoE4

S6c Table: Associations of diabetes drug classes with change in global cognition among individuals with diabetes who receive diabetes medications

			Metformin			Sulfonylurea			Insulin		
	Model	# cohorts	Estimate	SE	p-value	Estimate	SE	p-value	Estimate	SE	p-value
Including Dementia Cases	1	5	-0.00008	0.008	0.993	-0.012	0.006	0.073	-0.006	0.007	0.402
	2	5	-0.001	0.009	0.905	-0.010	0.007	0.122	-0.007	0.008	0.339
	3	5	-0.001	0.009	0.868	-0.010	0.007	0.121	-0.008	0.008	0.312
Excluding Dementia Cases	1	5	-0.002	0.008	0.780	-0.008	0.006	0.236	-0.012	0.008	0.104
	2	5	-0.004	0.009	0.668	-0.007	0.007	0.294	-0.013	0.008	0.103
	3	5	-0.004	0.009	0.681	-0.008	0.007	0.250	-0.013	0.008	0.110

Model 1: age, sex and education

Model 2: further adjustment for physical activity, hypertension, CVD, stroke, total cholesterol, smoking, depression, and BMI

Model 3: Further adjustment for HbA1C/ fasting blood glucose /random state blood glucose and ApoE4

S6d Table: Associations of diabetes drug classes with brain MRI measures among individuals with diabetes who receive diabetes medications

				Metformin			Sulfonylurea			Insulin		
		**Outcome**	**# cohorts**	**Estimate**	**SE**	**p-value**	**Estimate**	**SE**	**p-value**	**Estimate**	**SE**	**p-value**
Including prevalent dementia	Model 1	TCBV	5	0.004	0.003	0.257	-0.008	0.003	**0.010**	-0.012	0.005	**0.009**
		HPV	5	0.00002	0.00001	0.0717	0.000002	0.00001	0.842	-0.00002	0.00001	0.057
		WMHV	6	-0.066	0.0535	0.215	-0.006	0.053	0.907	0.093	0.065	0.152
	Model 2	TCBV	5	0.003	0.003	0.406	-0.008	0.004	**0.023**	-0.010	0.005	**0.024**
		HPV	5	0.000012	0.00001	0.057	0.000001	0.00001	0.921	-0.00002	0.00001	0.072
		WMHV	6	-0.029	0.0519	0.571	-0.006	0.052	0.911	0.039	0.061	0.517
	Model 3	TCBV	5	0.001	0.004	0.775	-0.006	0.004	0.087	-0.011	0.005	**0.015**
		HPV	5	0.00002	0.00001	0.134	0.000004	0.00001	0.689	-0.00001	0.00002	0.482
		WMHV	6	-0.0169	0.0523	0.747	-0.011	0.052	0.832	0.053	0.062	0.401
excluding prevalent dementia	Model 1	TCBV	4	0.0004	0.005	0.941	-0.010	0.004	**0.012**	-0.006	0.007	0.430
		HPV	4	0.004	0.006	0.516	-0.0005	0.006	0.931	-0.009	0.007	0.204
		WMHV	5	-0.077	0.056	0.170	-0.0008	0.056	0.989	0.104	0.067	0.122
	Model 2	TCBV	4	-0.0002	0.005	0.961	-0.0081	0.005	0.106	-0.005	0.007	0.517
		HPV	4	0.001	0.007	0.874	-0.002	0.006	0.734	-0.011	0.008	0.144
		WMHV	5	-0.043	0.054	0.420	0.006	0.053	0.911	0.058	0.062	0.351
	Model 3	TCBV	4	0.001	0.005	0.839	-0.0081	0.005	0.106	-0.002	0.007	0.732
		HPV	4	-0.0004	0.007	0.958	-0.001	0.007	0.861	-0.012	0.009	0.176
		WMHV	5	-0.035	0.054	0.516	0.005	0.054	0.920	0.078	0.0642	0.226

TCBV=Total cerebral brain volume; HPV=Hippocampal volume; WMHV=White matter hyperintensity volume

Model 1 is adjusted for age and sex and interval between exam cycle and date of MRI

Model 2 is additionally adjusted for Physical activity, hypertension, CVD, stroke, total cholesterol, smoking, depression, and BMI

Model 3 is additionally adjusted for HbA1C/FBG/random BG, ApoE4
