# Supplementary material for: Association of metformin, sulfonylurea and insulin use with brain structure and function and risk of dementia and Alzheimer’s disease: Pooled analysis from 5 cohorts
Source: PLoS One. 2019 Feb 15;14(2):e0212293. doi: 10.1371/journal.pone.0212293 (PMC6377188; doi:10.1371/journal.pone.0212293)
Supplement: S7 Table — S7a Table: Associations of diabetes drug classes with incident dementia/AD among individuals with diabetes S7b Table: Associations of diabetes drug classes with incident dementia/AD among diabetic participants who are on medications (excluding those who are only on life-style change) S7c Table: Associations of diabetes drug classes with cognitive performance among individuals with diabetes S7d Table: Associations of diabetes drug classes with cognitive performance among individuals with diabetes who are on medications (excluding those who are only on life-style change) S7e Table: Associations of diabetes drug classes with change in cognitive performance among individuals with diabetes S7f Table: Associations of diabetes drug classes with change in cognitive performance among individuals with diabetes who are on medications (excluding those who are only on life-style change) S7g Table: Associations of diabetes drug classes (single or in combination) with MRI measures among individuals with diabetes S7h Table: Associations of diabetes drug classes (single or in combination) with MRI measures among individuals on diabetes medications. (PDF) [file pone.0212293.s007.pdf]

S7. Random effect meta-analyses

S7a Table: Associations of diabetes drug classes with incident dementia/AD among individuals with diabetes

			Metformin		Sulfonylurea		Insulin	
	**Outcome**	**# cohorts**	**HR (95% CI)**	**p-value**	**HR (95% CI)**	**p-value**	**HR (95% CI)**	**p-value**
Model 1	Incident AD	4	1.22 (0.60, 2.49)	0.584	0.91 (0.59, 1.41)	0.677	1.61 (0.90, 2.89)	0.112
	Incident Dementia	5	1.26 (0.87, 1.83)	0.230	0.97 (0.78, 1.22)	0.800	1.61 (1.23, 2.11)	**<0.001**
Model 2	Incident AD	4	1.47 (0.70, 3.09)	0.307	0.98 (0.60, 1.6)	0.927	1.42 (0.67, 3.00)	0.358
	Incident Dementia	5	1.38 (0.96, 1.99)	0.086	0.97 (0.77, 1.23)	0.828	1.56 (1.17, 2.08)	**0.002**
Model 3	Incident AD	4	1.30 (0.51, 3.33)	0.579	1.04 (0.62, 1.74)	0.871	1.28 (0.56, 2.93)	0.556
	Incident Dementia	5	1.38 (0.97, 1.98)	0.076	0.98 (0.77, 1.24)	0.853	1.58 (1.18, 2.12)	**0.002**
Model 4	Incident AD	4	1.33 (0.55, 3.24)	0.528	0.90 (0.52, 1.57)	0.712	1.24 (0.53, 2.88)	0.616
	Incident Dementia	5	1.42 (1.02, 1.98)	0.038	0.98 (0.77, 1.26)	0.894	1.54 (1.14, 2.07)	**0.005**

Model 1 is adjusted for age, sex and education

Model 2 is additionally adjusted for Physical activity, hypertension, CVD, stroke, total cholesterol, smoking, depression and BMI

Model 3 is additionally adjusted for HbA1C, ApoE4

Model 4 is additionally adjusted for eGFR

S7b Table: Associations of diabetes drug classes with incident dementia/AD among diabetic participants who are on medications (excluding those who are only on life-style change)

			Metformin		Sulfonylurea		Insulin	
	**Outcome**	**# cohorts**	**HR (95% CI)**	**p-value**	**HR (95% CI)**	**p-value**	**HR (95% CI)**	**p-value**
Model 1	Incident AD	4	1.17 (0.45, 3.03)	0.742	0.91 (0.52, 1.57)	0.726	1.52 (0.81, 2.84)	0.195
	Incident Dementia	5	1.16 (0.68, 1.96)	0.589	0.73 (0.55, 0.96)	**0.025**	1.41 (1.05, 1.90)	**0.021**
Model 2	Incident AD	4	1.42 (0.46, 4.37)	0.537	0.75 (0.28, 2.03)	0.572	1.46 (0.58, 3.72)	0.424
	Incident Dementia	5	1.31 (0.72, 2.38)	0.376	0.69 (0.47, 1.02)	0.062	1.50 (1.09, 2.06)	**0.014**
Model 3	Incident AD	4	1.25 (0.37, 4.22)	0.722	0.68 (0.20, 2.33)	0.539	1.39 (0.54, 3.53)	0.494
	Incident Dementia	5	1.34 (0.73, 2.44)	0.345	0.68 (0.44, 1.06)	0.090	1.55 (1.12, 2.15)	**0.009**
Model 4	Incident AD	4	0.75 (0.14, 3.98)	0.733	0.31 (0.05, 1.94)	0.210	1.07 (0.36, 3.15)	0.907
	Incident Dementia	5	1.36 (0.75, 2.49)	0.311	0.63 (0.32, 1.24)	0.179	1.49 (1.07, 2.07)	**0.018**

Model 1 is adjusted for age, sex and education
Model 2 is additionally adjusted for Physical activity, hypertension, CVD, stroke, total cholesterol, smoking, depression and BMI
Model 3 is additionally adjusted for HbA1C, ApoE4
Model 4 is additionally adjusted for eGFR

S7c Table: Associations of diabetes drug classes with cognitive performance among individuals with diabetes

			Metformin			Sulfonylurea			Insulin		
	**Outcome**	**# cohorts**	**Estimate**	**SE**	**p-value**	**Estimate**	**SE**	**p-value**	**Estimate**	**SE**	**p-value**
Model 1	Global cognition	6	-0.045	0.039	0.254	-0.075	0.029	**0.010**	-0.117	0.077	0.125
	Executive function (trails B-A)	3	-0.022	0.065	0.738	-0.079	0.044	0.070	-0.051	0.107	0.631
	Word list - delayed	5	-0.013	0.029	0.663	-0.028	0.037	0.443	-0.041	0.063	0.514
	Word list - combined	4	-0.049	0.044	0.273	-0.019	0.041	0.644	-0.003	0.059	0.956
	Paragraph recall - delayed	3	0.042	0.034	0.227	-0.012	0.040	0.760	-0.076	0.049	0.118
	Paragraph recall - combined	3	0.057	0.034	0.098	0.001	0.041	0.971	-0.074	0.049	0.127
Model 2	Global cognition	6	-0.052	0.033	0.118	-0.074	0.034	**0.032**	-0.066	0.067	0.326
	Executive function (trails B-A)	3	-0.027	0.059	0.651	-0.072	0.045	0.113	-0.074	0.068	0.273
	Word list - delayed	5	-0.030	0.030	0.317	-0.021	0.046	0.644	-0.003	0.071	0.961
	Word list - combined	4	-0.040	0.046	0.379	-0.005	0.050	0.926	0.069	0.059	0.245
	Paragraph recall - delayed	3	0.019	0.036	0.593	-0.076	0.069	0.272	-0.036	0.053	0.500
	Paragraph recall - combined	3	0.034	0.036	0.340	-0.079	0.083	0.343	-0.029	0.053	0.580
Model 3	Global cognition	6	-0.040	0.040	0.324	-0.045	0.030	0.137	-0.020	0.053	0.706
	Executive function (trails B-A)	3	0.001	0.071	0.990	-0.050	0.049	0.305	0.034	0.097	0.731
	Word list - delayed	5	-0.027	0.031	0.379	-0.015	0.043	0.735	0.000	0.056	0.996
	Word list - combined	4	-0.050	0.048	0.292	-0.011	0.051	0.832	0.075	0.060	0.215
	Paragraph recall - delayed	3	0.024	0.037	0.508	-0.098	0.092	0.283	0.002	0.058	0.974
	Paragraph recall - combined	3	0.035	0.036	0.329	-0.106	0.108	0.327	0.007	0.058	0.902

Model 1: Age, sex, education and interval between exam cycle and the cognitive assessment

Model 2: Model 1 + Physical activity, hypertension, CVD, stroke, total cholesterol, smoking, depression, BMI

Model 3: Model 2+HbA1C/ fasting blood glucose /random state blood glucose and ApoE4

S7d Table: Associations of diabetes drug classes with cognitive performance among individuals with diabetes who are on medications (excluding those who are only on life-style change)

			Metformin			Sulfonylurea			Insulin		
	**Outcome**	**# cohorts**	**Estimate**	**SE**	**p-value**	**Estimate**	**SE**	**p-value**	**Estimate**	**SE**	**p-value**
Model 1	Global cognition	6	0.021	0.072	0.770	-0.050	0.045	0.266	-0.086	0.069	0.212
	Executive function (trails B-A)	3	0.067	0.050	0.179	-0.017	0.049	0.736	-0.028	0.065	0.666
	Word list - delayed	5	0.003	0.052	0.955	-0.011	0.049	0.826	-0.014	0.076	0.857
	Word list - combined	4	-0.056	0.051	0.274	-0.018	0.010	0.085	0.025	0.065	0.699
	Paragraph recall - delayed	3	0.119	0.045	**0.008**	0.014	0.044	0.742	-0.065	0.051	0.203
	Paragraph recall - combined	3	0.111	0.045	**0.013**	0.003	0.057	0.961	-0.077	0.051	0.129
Model 2	Global cognition	6	-0.009	0.061	0.880	-0.060	0.038	0.119	-0.059	0.046	0.201
	Executive function (trails B-A)	3	0.050	0.052	0.330	-0.014	0.051	0.787	-0.017	0.061	0.777
	Word list - delayed	5	-0.017	0.044	0.694	-0.020	0.048	0.679	0.012	0.075	0.874
	Word list - combined	4	-0.074	0.051	0.147	0.002	0.010	0.870	0.077	0.062	0.217
	Paragraph recall - delayed	3	0.085	0.048	0.078	-0.079	0.094	0.402	-0.010	0.055	0.857
	Paragraph recall - combined	3	0.070	0.048	0.142	-0.081	0.098	0.409	-0.017	0.055	0.752
Model 3	Global cognition	6	-0.015	0.055	0.784	-0.040	0.035	0.246	-0.026	0.042	0.532
	Executive function (trails B-A)	3	0.051	0.052	0.330	-0.001	0.052	0.978	0.032	0.067	0.632
	Word list - delayed	5	-0.029	0.039	0.463	-0.017	0.042	0.688	0.023	0.061	0.713
	Word list - combined	4	-0.084	0.052	0.106	-0.023	0.053	0.666	0.088	0.064	0.170
	Paragraph recall - delayed	3	0.067	0.048	0.165	-0.102	0.107	0.342	0.017	0.060	0.780
	Paragraph recall - combined	3	0.047	0.048	0.332	-0.111	0.117	0.344	0.011	0.059	0.856

Model 1: Age, sex, education and interval between exam cycle and the cognitive assessment

Model 2: Model 1 + Physical activity, hypertension, CVD, stroke, total cholesterol, smoking, depression, BMI

Model 3: Model 2+HbA1C/ fasting blood glucose /random state blood glucose and ApoE4

S7e Table: Associations of diabetes drug classes with change in cognitive performance among individuals with diabetes

			Metformin*			Sulfonylurea			Insulin		
	Model	# cohorts	Estimate	SE	p-value	Estimate	SE	p-value	Estimate	SE	p-value
Including prevalent dementia	1	5	0.008	0.012	0.538	-0.010	0.005	0.058	-0.007267	0.008	0.383
	2	5	0.006	0.013	0.653	-0.011	0.006	0.055	-0.00888	0.007	0.214
	3	5	0.005	0.013	0.715	-0.011	0.006	**0.046**	-0.010609	0.007	0.146
Excluding prevalent dementia	1	5	0.005	0.012	0.690	-0.007	0.006	0.255	-0.012377	0.007	0.083
	2	5	0.004	0.013	0.780	-0.008	0.007	0.247	-0.013139	0.007	0.073
	3	5	0.002	0.013	0.859	-0.009	0.007	0.189	-0.014457	0.007	0.054

Model 1: age, sex and education

Model 2: further adjustment for physical activity, hypertension, CVD, stroke, total cholesterol, smoking, depression, and BMI

Model 3: Further adjustment for HbA1C/ fasting blood glucose /random state blood glucose and ApoE4

S7f Table: Associations of diabetes drug classes with change in cognitive performance among individuals with diabetes who are on medications (excluding those who are only on life-style change)

			Metformin			Sulfonylurea			Insulin		
	Outcome	# cohorts	Estimate	SE	p-value	Estimate	SE	p-value	Estimate	SE	p-value
Model 1	Including Dementia Cases	5	0.005	0.013	0.688	-0.012	0.008	0.104	-0.006	0.008	0.402
	Excluding Dementia Cases	5	0.003	0.013	0.841	-0.010	0.010	0.331	-0.012	0.008	0.113
Model 2	Including Dementia Cases	5	0.003	0.014	0.807	-0.011	0.009	0.210	-0.008	0.008	0.335
	Excluding Dementia Cases	5	-0.0002	0.014	0.990	-0.007	0.011	0.490	-0.013	0.008	0.108
Model 3	Including Dementia Cases	5	0.002	0.014	0.891	-0.011	0.008	0.195	-0.008	0.008	0.308
	Excluding Dementia Cases	5	-0.0006	0.014	0.969	-0.008	0.011	0.459	-0.013	0.008	0.114

Model 1: age, sex and education

Model 2: further adjustment for physical activity, hypertension, CVD, stroke, total cholesterol, smoking, depression, and BMI

Model 3: Further adjustment for HbA1C/ fasting blood glucose /random state blood glucose and ApoE4

S7g Table: Associations of diabetes drug classes (single or in combination) with MRI measures among individuals with diabetes

				Metformin			Sulfonylurea			Insulin		
		Outcome	# cohorts	Estimate	SE	p-value	Estimate	SE	p-value	Estimate	SE	p-value
Including prevalent dementia	Model 1	TCBV	5	-0.003	0.004	0.490	-0.010	0.008	0.193	-0.0176	0.020	0.370
		HPV	5	-0.0006	0.003	0.827	-0.001	0.003	0.624	-0.002	0.003	0.565
		WMHV	6	0.062	0.038	0.105	0.077	0.044	0.083	0.149	0.077	0.053
	Model 2	TCBV	5	-0.002	0.003	0.546	-0.008	0.007	0.286	-0.017	0.021	0.411
		HPV	5	-0.001	0.003	0.621	-0.001	0.003	0.670	-0.002	0.003	0.482
		WMHV	6	0.037	0.038	0.339	0.046	0.045	0.301	0.072	0.088	0.416
	Model 3	TCBV	5	-0.001	0.003	0.737	-0.006	0.005	0.169	-0.010	0.014	0.487
		HPV	5	-0.0009	0.003	0.735	-0.0007	0.003	0.806	-0.001	0.003	0.701
		WMHV	6	0.030	0.034	0.445	0.034	0.045	0.448	0.040	0.061	0.510
excluding prevalent dementia	Model 1	TCBV	4	-0.028	0.059	0.633	-0.364	0.251	0.148	-0.789	0.514	0.125
		HPV	4	-0.002	0.005	0.714	-0.006	0.006	0.282	-0.011	0.007	0.114
		WMHV	5	0.047	0.040	0.241	0.078	0.046	0.089	0.157	0.088	0.074
	Model 2	TCBV	4	0.004	0.004	0.371	-0.303	0.262	0.248	-0.885	0.546	0.105
		HPV	4	-0.004	0.005	0.435	-0.005	0.006	0.435	-0.013	0.007	0.093
		WMHV	5	0.019	0.040	0.640	0.049	0.046	0.287	0.082	0.094	0.383
	Model 3	TCBV	4	-0.002	0.004	0.640	-0.138	0.158	0.383	-0.737	0.437	0.092
		HPV	4	-0.002	0.005	0.686	-0.003	0.006	0.670	-0.011	0.008	0.183
		WMHV	5	0.012	0.041	0.775	0.041	0.046	0.374	0.070	0.080	0.389

TCBV=Total cerebral brain volume; HPV=Hippocampal volume; WMHV=White matter hyperintensity volume

Model 1 is adjusted for age and sex and interval between exam cycle and date of MRI

Model 2 is additionally adjusted for Physical activity, hypertension, CVD, stroke, total cholesterol, smoking, depression, and BMI

Model 3 is additionally adjusted for HbA1C/FBG/random BG, ApoE4

S7h Table: Associations of diabetes drug classes (single or in combination) with MRI measures among individuals on diabetes medications

				Metformin			Sulfonylurea			Insulin		
		Outcome	# cohorts	Estimate	SE	p-value	Estimate	SE	p-value	Estimate	SE	p-value
Including prevalent dementia	Model 1	TCBV	5	0.003	0.005	0.511	-0.008	0.004	**0.040**	-0.013	0.014	0.357
		HPV	5	0.0005	0.003	0.852	-0.0001	0.003	0.964	-0.001	0.003	0.691
		WMHV	6	-0.066	0.054	0.211	-0.006	0.054	0.907	0.093	0.065	0.152
	Model 2	TCBV	5	0.003	0.004	0.525	-0.008	0.004	0.063	-0.012	0.017	0.473
		HPV	5	-0.00008	0.003	0.978	-0.0004	0.003	0.902	-0.001	0.003	0.634
		WMHV	6	-0.029	0.052	0.571	-0.020	0.075	0.787	0.039	0.061	0.517
	Model 3	TCBV	5	0.001	0.004	0.808	-0.006	0.004	0.147	-0.012	0.013	0.370
		HPV	5	-0.0002	0.003	0.932	-0.0001	0.003	0.972	-0.001	0.003	0.708
		WMHV	6	-0.0169	0.052	0.747	-0.030	0.076	0.693	0.051	0.069	0.461
excluding prevalent dementia	Model 1	TCBV	4	0.0004	0.006	0.946	-0.010	0.005	0.047	-0.611	0.423	0.149
		HPV	4	0.004	0.006	0.535	-0.0006	0.006	0.931	-0.009	0.007	0.228
		WMHV	5	-0.077	0.056	0.170	-0.0002	0.069	0.998	0.104	0.067	0.122
	Model 2	TCBV	4	-0.0002	0.006	0.969	-0.110	0.189	0.560	-0.773	0.478	0.106
		HPV	4	0.0008	0.007	0.910	-0.002	0.007	0.731	-0.011	0.008	0.161
		WMHV	5	-0.043	0.054	0.420	-0.007	0.082	0.932	0.056	0.071	0.432
	Model 3	TCBV	4	0.001	0.006	0.862	-0.008	0.006	0.170	-0.726	0.4506	0.107
		HPV	4	-0.0006	0.007	0.929	-0.001	0.007	0.859	-0.012	0.009	0.192
		WMHV	5	-0.035	0.054	0.517	-0.001	0.068	0.987	0.071	0.093	0.445

TCBV=Total cerebral brain volume; HPV=Hippocampal volume; WMHV=White matter hyperintensity volume

Model 1 is adjusted for age and sex and interval between exam cycle and date of MRI

Model 2 is additionally adjusted for Physical activity, hypertension, CVD, stroke, total cholesterol, smoking, depression, and BMI

Model 3 is additionally adjusted for HbA1C/FBG/random BG, ApoE4
